# Opening the Random Forest Black Box of ^1^H NMR Metabolomics Data by the Exploitation of Surrogate Variables

**DOI:** 10.3390/metabo13101075

**Published:** 2023-10-13

**Authors:** Soeren Wenck, Thorsten Mix, Markus Fischer, Thomas Hackl, Stephan Seifert

**Affiliations:** 1 Institute of Food Chemistry, Hamburg School of Food Science, University of Hamburg, Grindelallee 117, 20146 Hamburg, Germanymarkus.fischer@uni-hamburg.de (M.F.); thomas.hackl@uni-hamburg.de (T.H.); 2 Institute of Organic Chemistry, University of Hamburg, Martin-Luther-King-Platz 6, 20146 Hamburg, Germany; thorsten.mix@uni-hamburg.de

**Keywords:** classification, characterization, nuclear magnetic resonance spectroscopy, random forest, variable selection, variable relations, machine learning, chemometrics, surrogate minimal depth, truffles

## Abstract

The untargeted metabolomics analysis of biological samples with nuclear magnetic resonance (NMR) provides highly complex data containing various signals from different molecules. To use these data for classification, e.g., in the context of food authentication, machine learning methods are used. These methods are usually applied as a black box, which means that no information about the complex relationships between the variables and the outcome is obtained. In this study, we show that the random forest-based approach surrogate minimal depth (SMD) can be applied for a comprehensive analysis of class-specific differences by selecting relevant variables and analyzing their mutual impact on the classification model of different truffle species. SMD allows the assignment of variables from the same metabolites as well as the detection of interactions between different metabolites that can be attributed to known biological relationships.

## 1. Introduction

Metabolomics is the research field that aims at the comprehensive analysis of metabolites, which are small molecules (<1500 Da) within biological organisms. Metabolites take part in cellular regulatory processes and are influenced by both endogenous factors such as the genotype and exogeneous factors such as climate, soil composition, distance to large bodies of waters, and fertilization [1]. Thus, the metabolome is the best representation of the phenotype [2]. Since there is no approach that can capture the entire metabolome, various combinations of extraction and measurement techniques have been introduced through which different parts of the metabolome can be analyzed [3]. Many of these analytical methods are based on nuclear magnetic resonance (NMR) and mass spectrometry (MS) platforms [4,5,6,7]. 

^1^H NMR combines highly repeatable and reproducible non-destructive data acquisition, simultaneous structural elucidation and quantitative analysis of compounds. However, interpreting NMR spectra of biological samples is difficult, since they contain hundreds of signals from several dozens of metabolites [7,8,9,10,11]. For this reason, assigning signals to specific molecules is not straightforward and usually requires individual strategies. A number of databases and tools are available, such as the Human Metabolome Database (HMDB), the Biological Magnetic Resonance Database (BMRB) or the Chenomx software [12]. In addition to some inherent errors that can occur in any database, experimental conditions such as solvent, pH, or ionic strength have a huge impact on chemical shifts and make the exclusive use of databases difficult, leading to unreliable assignments. Besides the standard 2D NMR methods, such as TOCSY or HSQC, some classical experiments, such as *J*-resolved NMR or 1D methods, such as selective TOCSY or NOESY have gained new popularity [13]. The combination of different experiments increases the likelihood of identifying additional metabolites, and the combination of NMR and MS is a promising approach to identifying compounds of interest because these analytical techniques offer complementary information. Recently published cheminformatics combinations of NMR and MS are the NMR/MS translator [14] and the SUMMIT MS/NMR method [15]. The correlation between NMR and MS data can be established when these techniques are used in combination with liquid chromatography, which has been demonstrated through approaches such as parallel dynamic NMR/LC-MS spectroscopy (NMR/LC-MS PDS) [16] or the Semi-automatic COrrelation analysis for REliable metabolite IDentification (SCORE-metabolite-ID) [17]. Typically, spike-in experiments with either purchased or synthesized reference compounds are performed on the mixture samples to verify the proposed structures. 

NMR data can be analyzed using a technique called Statistical Total Correlation Spectroscopy (STOCSY) to detect correlated NMR signals based on structural connectivity or intermolecular correlations resulting from the connectivity of metabolic pathways in biological systems [18]. However, STOCSY and other statistics-based approaches require large sample sets for analysis and cannot distinguish between different types of correlation. Statistical heterospectroscopy (SHY) is another approach that is based on STOCSY but uses a combination of NMR and MS data [19].

The analysis of NMR metabolomics data is usually performed by either fitting patterns of signals from expected metabolites to spectral regions within the data or binning [1,7,20]. The latter is usually applied to aligned spectra to reduce the chemical shift variety and to achieve comparability among different spectra [21]. Since NMR data sets are high-dimensional, meaning that they contain many variables from comparatively few samples, multivariate approaches have to be applied for data analysis [22]. The popular unsupervised approach principal component analysis (PCA) creates latent variables by linear combinations of the original variables. These principal components are focused on the main variances of the data and can enable the identification of groups with similar patterns [23,24,25]. In contrast to unsupervised approaches, supervised machine learning algorithms such as support vector machines (SVM) [26], artificial neural networks (ANN) [27], and random forests (RF) include the group affiliation of samples in the analysis and train classification models based on specific class differences.

RF is a non-parametric ensemble learning algorithm based on multiple binary decision trees that offers many advantages for application to high-dimensional data, such as the inherent independent validation [28,29]. This validation is based on the fact that each of the decision trees is trained on a different fraction of the samples, the so-called bootstrap samples, while the respective remaining samples are used to generate independent out-of-bag errors. Another advantage of RF is that it can also be used to generate variable importance scores. These scores are, for example, based on the decrease of accuracy obtained by the permutation of a variable or on the decrease of Gini impurity calculated by the summarized Gini gains, a variable is contributing to the RF. Variable selection methods use these importance scores to separate important from unimportant variables, and various approaches that differ in the way in which they define the threshold between important and unimportant variables have been developed. Boruta creates shadow variables by random permutation and evaluates whether the real variables generally show higher importance scores than the highest scores of the shadow variables [30]. Surrogate Minimal Depth (SMD) is a variable importance score and selection approach that incorporates variable relations into the selection process [31]. This is achieved by the combination of minimal depth [32], an importance measure based on the first appearance of variables in decision trees, with surrogate variables, which were originally introduced by Breimann et al. [28] for the compensation of missing variables. SMD thus determines the variable importance measure not only by considering primary split variables but also surrogate variables. In addition to variable selection, SMD can also be applied to calculate the relation parameter *mean adjusted agreement*, analyzing the mutual impact of the variables on the random forest model. This relation parameter, which has recently been further developed to also analyze qualitative variables [33], enables a comprehensive analysis of the interplay of the relevant variables. It has been successfully applied in various fields and to different types of data, including gene expression [31], surface-enhanced Raman scattering [34,35], FT-NIR [5], and LC MS data [36], as well as to analyze relations across the latter two analytical techniques [37].

Here, we apply SMD to ^1^H NMR metabolomics data for the first time and show that it can reveal various relationships between predictor variables and outcome, as well as between predictor variables. More precisely, buckets containing information from the same signals and molecules can be identified, and meaningful biological relations between different metabolites can be determined and utilized for the investigation of specific class differences. As a model data set, we use data from truffle samples as the truffle species show a clear distinction and, thus, a comparatively simple interpretation of the selected markers and observed differences is possible [38]. Due to limited harvest periods, difficult cultivation, and their unique aromatic properties, truffles are one of the most expensive foods and, hence, prone to food fraud [39,40].

## 2. Materials and Methods

### 2.1. Samples and Data Acquisition

The ^1^H NMR data set used in this study contained 80 samples from five different *Tuber* species (see Table 1) and is provided in Table S1. For detailed information about the measurement and preprocessing of the data, please refer to Mix et al. [38]. However, the data utilized here adopted a bucket width of 0.01 ppm, whereas Mix et al. opted for a width of 0.03 ppm. In addition to the ^1^H NMR measurement, every sample was analyzed with ^1^H-^1^H TOCSY. The measurement was conducted with the *dipsi2esgpph* (Bruker notation) pulse sequence. Homonuclear Hartman-Hahn transfer using *DIPSI2* (Bruker notation) sequence for mixing was performed. The data were collected with a spectral width of 4401.4 Hz. The spin-locking field of 8.$\overset{-}{3}$ KHz was generated with a 30 μs pulse at a power of −2.5 dB. Eight scans per increment in a matrix of 2048 × 256 were obtained with a mixing time of 60 ms, and the data were zero-filled to 2048 × 512. To generate phase-sensitive data, the States-*TPPI* phase cycling was used. The data were processed with a *QSINE* function in both dimensions and a Sine Bell Shift (SSB) of 2. The parameter set *dipsi2esgpph* (Bruker notation) was applied in accordance with Shaka et al. for water suppression [41].

### 2.2. Identification of Truffle Metabolites

The identification of metabolites was carried out according to Mix et al. [38] by column chromatographic fractionation of the mixture and subsequent analysis of the fractions by NMR and MS techniques. The NMR and MS signals were correlated manually or using the SCORE-metabolite-ID app [17]. For the verification of proposed structures, spike-in experiments were performed in which 10 to 200 μg of a specific metabolite was added to one of the sample fractions containing the corresponding metabolite. The mixtures were remeasured with the pulse program noesygppr1d (Bruker notation) at 300 K. For visual clarity, the measurements were conducted at 400 MHz or 600 MHz (Ribonate) and with 32 or 64 scans with TMSP as an internal standard. An increase in the signal intensity confirmed the spiked metabolite in the spectrum [42].

### 2.3. Software and Data Analysis 

Data acquisition was performed with Topspin (version 4.0.94) and bucketing with Aurelia Amix (version 3.9.15). The software R (version 3.6.3) and the R packages ranger (version 0.14.1, CRAN) for RF classification [43], mdatools (version 0.12.0, CRAN) for PCA [44], Pomona (version 1.0.1, https://github.com/silkeszy/Pomona, accessed on 11 October 2023) for Boruta variable selection [45], and SurrogateMinimalDepth (version 0.2.0, https://github.com/StephanSeifert/SurrogateMinimalDepth, accessed on 11 October 2023) for SMD variable selection and relation analysis were used [31]. Figures were created with ggplot2 (version 3.4.0, CRAN) [46] and heatmaps with pheatmap (version 1.0.12, CRAN, https://CRAN.R-project.org/package=pheatmap, accessed on 11 October 2023) [47]. 

The RF approaches were applied in classification mode with the parameters listed in Table 2. Due to the imbalance of the classes, the samples were weighted accordingly using the parameter case.weights. The variable relation analysis was performed on variables selected by Boruta and SMD, analyzing relationships that were assigned to the same signal and those that corresponded to different signals and metabolites. For the latter, a hierarchical cluster analysis with Euclidean distance measure and Ward’s algorithm [48] was applied. For the clarity of this analysis, the variables of the same signals covering multiple buckets were reduced to one representative each, which was chosen by the lowest surrogate minimal depth value, i.e., the highest importance. In addition, the variables that could not be identified clearly were also removed from this analysis. 

## 3. Results and Discussion

### 3.1. Classification of Truffle Samples

**Figure 1:**
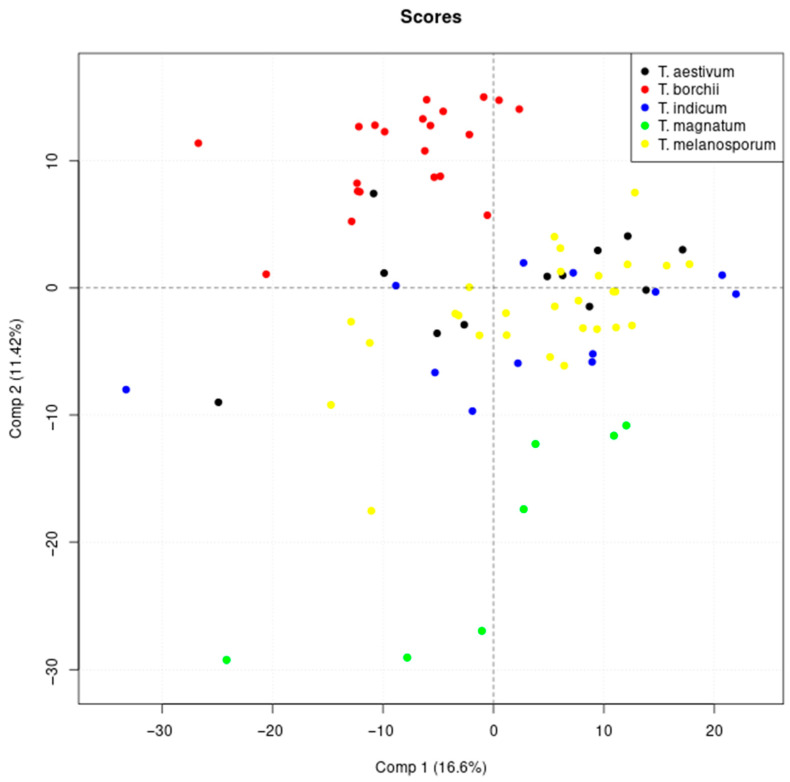
Results of the principal component analysis: Scores of the first and second principal components are shown.

The main objective of this study was to open the black box of the ^1^H NMR metabolome by the application of random forest-based approaches. For this, a data set with clear distinction between classes was needed and we applied random forest on the truffle data containing 80 samples from five different species to verify whether this was the case. The confusion matrix of the classification results is shown in Table 3, showing an accuracy of 100% confirming the prerequisites formulated above and the previous classification results that were obtained by support vector machines [38]. These clear differences between the truffle species are only partially evident from the results of the unsupervised principal component analysis, demonstrating that supervised approaches should be applied for classification (see Figure 1 and Figure S1).

### 3.2. Bucket Assignment for Truffle Metabolites 

In principle, knowledge of the underlying metabolites is not necessary for classification. However, it is essential for biological interpretation. We used a metabolite identification procedure described in [38]. Identification was carried out both independently of the SMD results, in particular by using the SCORE-metabolite-ID app and further NMR experiments, and especially when relationships between different buckets resulted from the SMD analysis. A total of 35 metabolites were identified. Based on fractionation by LC-MS-NMR correlation, the identities of all metabolites could be verified by spike-in experiments of the single fractions. Furthermore, as data from total extracts were used for classification and SMD analysis, spike-in experiments were also performed on the total extracts to clearly assign the corresponding buckets. The NMR spectra from these spike-in experiments are shown in Figures S10–S34. 23 of these metabolites were considered in the SMD analysis. They included amino acids (aspartic acid, asparagine, arginine, isoleucine, glutamic acid, glutamine, histidine, leucine, lysine, proline, threonine, tryptophan, and valine), carbohydrates (trehalose and ribonate), organic acids (citric, fumaric, and malic acid), uridine 5’-diphosphate-*N*-acetylglucoseamine (UDP-GlcNAc), betaine, choline-O-sulfate, and glycerophosphorylcholine (GPC). 

### 3.3. Variable Selection

**Figure 2:**
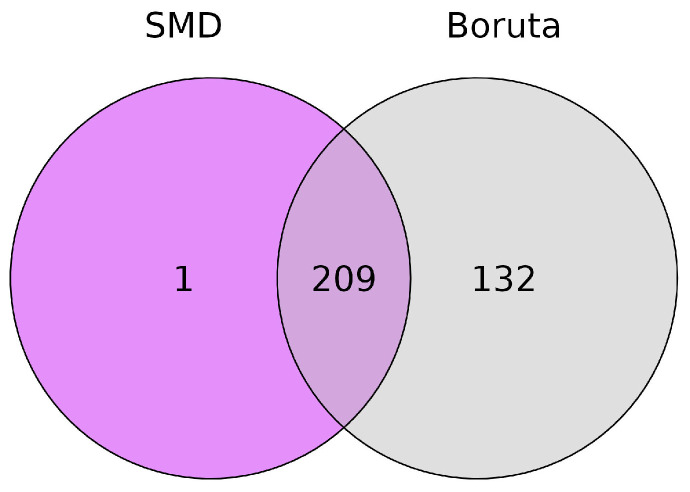
Venn diagram showing the overlap of variables selected by SMD and Boruta.

The first step on the way from black box classification to the comprehensive characterization of the metabolites involved is the selection of relevant variables by variable selection approaches. For this, the two approaches SMD and Boruta were applied, selecting 210 and 341 variables, respectively. The selected variables are listed in Table S2. Many variables with high importance could be assigned to organic or amino acids and carbohydrates, e.g., fumaric acid, lysine, and trehalose. The latter is a major fungal carbohydrate in ectomycorrhizal fungi such as truffles that are, in addition to their role in carbohydrate storage, involved in various cellular processes not directly related to carbohydrate metabolism [49]. Figure 2 shows the overlap of the selected variables of the two approaches: SMD selected only one variable that was not selected by Boruta, while Boruta selected additional 132 variables. In principle, the two selection approaches have very different objectives: Boruta evaluates the importance of a variable individually, while SMD includes variable relations into the selection process analyzing their mutual impact. Hence, the variables that were selected only by Boruta should show comparatively low relations to other variables. This is confirmed when comparing the variable relations of both methods in Figures S2 and S3, because the variables selected only by Boruta show almost no relation to other variables. To further investigate the variables that contribute mutual information, the relationship parameter mean adjusted agreement generated by SMD is examined in more detail in the following section. 

### 3.4. Analysis of Variable Relations 

The obtained relations between the selected variables could be attributed to different causes. For clarity, these are discussed separately in the following sections. 

#### 3.4.1. Relations of Variables Containing the Same Signals 

**Figure 3:**
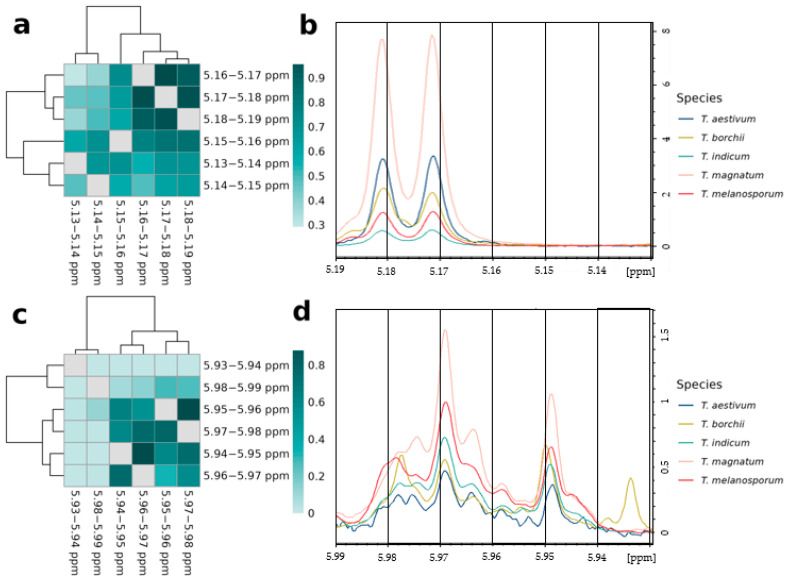
Analysis of adjacent variables from the same signals: Shown are heatmaps of mean adjusted agreement values and parts of the NMR spectra for the spectral regions between 5.13 and 5.19 ppm (**a**,**b**) and between 5.93 and 5.99 ppm (**c**,**d**). For the latter, one representative spectrum for each truffle species is shown and the black vertical lines show the limits of the buckets. For the heatmaps, cluster analysis with Euclidean distance measure and Ward’s algorithm was applied.

We frequently observed neighboring buckets with very high *mean adjusted agreement* values, often above 0.9. In Figure 3, this is shown exemplarily for the two spectral regions between 5.13 and 5.19 ppm and between 5.93 and 5.99 ppm, which were assigned to trehalose (see Figure S26) and UDP-GlcNAc (see Figure S12), respectively. It is obvious that the high mean adjusted agreement values are caused by the same respective multiplet signal that is present in multiple buckets. The linewidth of NMR signals is approximately between 0.7 and 3 Hz. A bucket size of 0.01 ppm corresponds exactly to 4 Hz. Thus, a single line can either lie exactly in one bucket or cross the bucket boundary into two adjacent buckets. Coupling constants range from 0 to 18 Hz. Thus, two lines belonging to the same signal may be separated by one to two buckets. Trehalose shows a doublet between 5.16 and 5.19 ppm and a coupling constant of 3.9 Hz (Figure 3b). As both lines are exactly on the bucket boundaries, the doublet extends over three buckets, which are highly related to each other and provide similar information to the classification model (Figure 3a), while the other buckets between 5.13 and 5.16 ppm mainly contain noise and show comparatively low relations. Similarly, the doublet of UDP-GlcNAc between 5.94 and 5.98 ppm, with a coupling constant of 8.1 Hz (Figure 3d), causes very strong relations of the respective buckets with each other (Figure 3c), while comparatively low relations occur to the buckets between 5.93 and 5.94 ppm as well as 5.98 and 5.99 ppm. 

**Figure 4:**
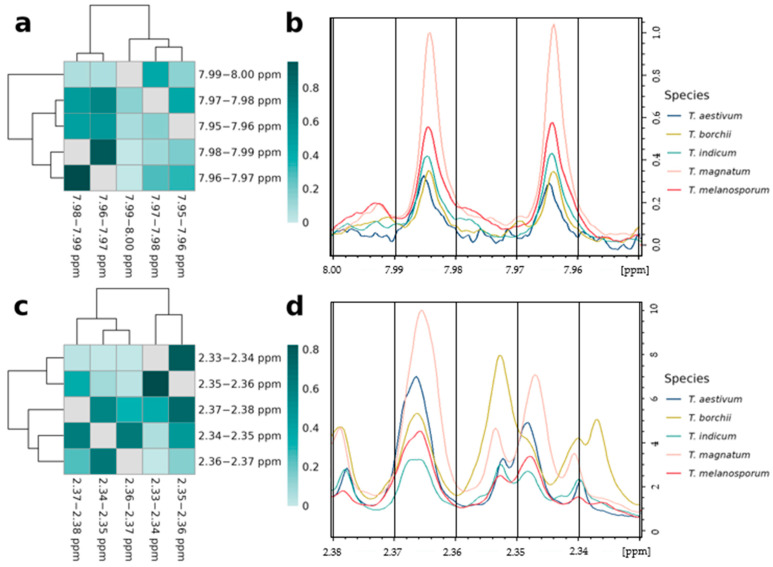
Analysis of close-by variables from the same signals: Heatmaps of mean adjusted agreement values and parts of the NMR spectra for the spectral regions between 7.95 and 8.00 ppm (**a**,**b**) and between 2.33 and 2.37 ppm (**c**,**d**). For the latter, one representative spectrum for each truffle species is shown and the black vertical lines show the limits of the buckets. For the heatmaps, cluster analysis with Euclidean distance measure and Ward’s algorithm was applied.

We also observed variables with high mean adjusted agreement values that were not directly next to each other, but still very close together. This is shown by the two spectral regions between 7.95 and 8.00 ppm and 2.33 and 2.37 ppm in Figure 4. In the first region, there is a strong relation between the buckets at 7.98–7.99 ppm and 7.96–7.97 ppm, while the relation with the other variables in this area, including the variable between them at 7.97–7.98 ppm, is much weaker (Figure 4a). The reason for this is that the two subpeaks of a doublet assigned to UDP-GlcNAc (see Figure S12) populate exactly one bucket and are separated by a coupling constant of 8 Hz. The variable at 7.97–7.98 ppm does not contain any signal intensity from this doublet (Figure 4b). 

For the spectral region between 2.33 and 2.37 ppm, two different clusters are built: the variables at 2.34–2.35 ppm and 2.36–2.37 ppm, assigned to a doublet of glutamic acid (see Figure S33), are strongly related to each other, while the other variables in the other cluster at 2.36–2.37 ppm, 2.34–2.35 ppm, and 2.37–2.38 ppm show slightly lower values for the relation parameter (Figure 4c). Hence, the glutamic acid doublet is overlapping with a second doublet, which is most pronounced at the buckets at 2.36–2.37 ppm and 2.34–2.35 ppm. That signals of two different metabolites are present here is also evident from the fact that the intensities of the truffle species are different: in the buckets 2.34–2.35 ppm and 2.36–2.37 ppm, the spectrum of *T. magnatum* is most intense, while *T. borchii* shows the most intensive peaks at 2.33–2.34 ppm and 2.35–2.36 ppm (Figure 4d).

#### 3.4.2. Relations of Variables from the Same Metabolites

**Figure 5:**
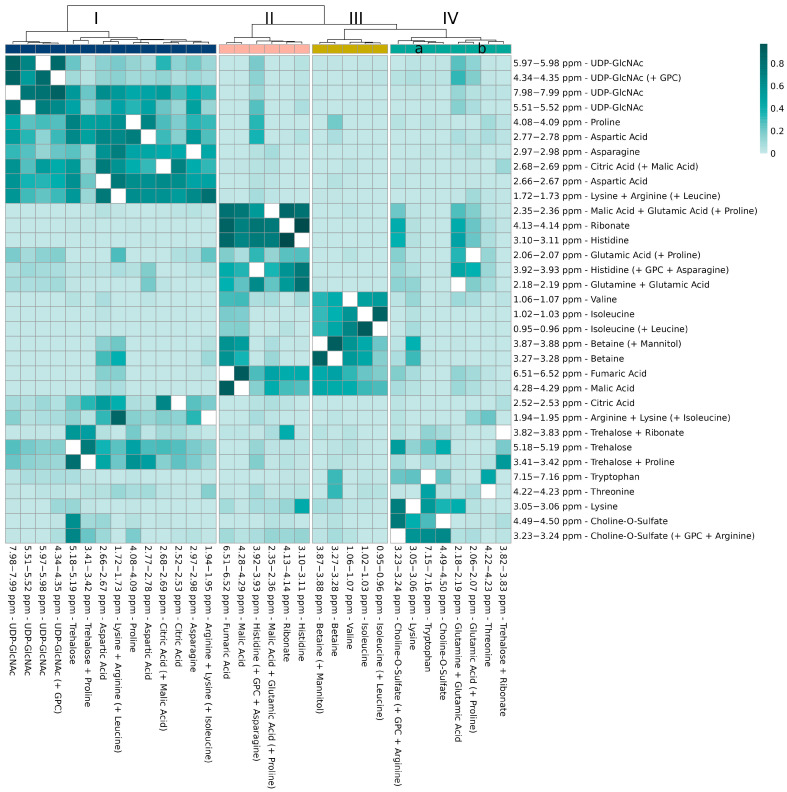
Result of the relation analysis of the identified variables. For the hierarchical cluster analysis, Euclidean distances and Ward’s algorithm were applied and the clusters are labeled with I—IVa/b. The variables are labelled with the assigned metabolites, whereby the assignments, which play a rather minor role for the classification due to the relationship analysis, are shown in brackets (see discussion in Section 3.4.2). Abbreviations: GPC—Glycerophosphorylcholine; UDP-GlcNAc—uridine 5′-diphosphate-*N*-acetylglucoseamine.

**Figure 6:**
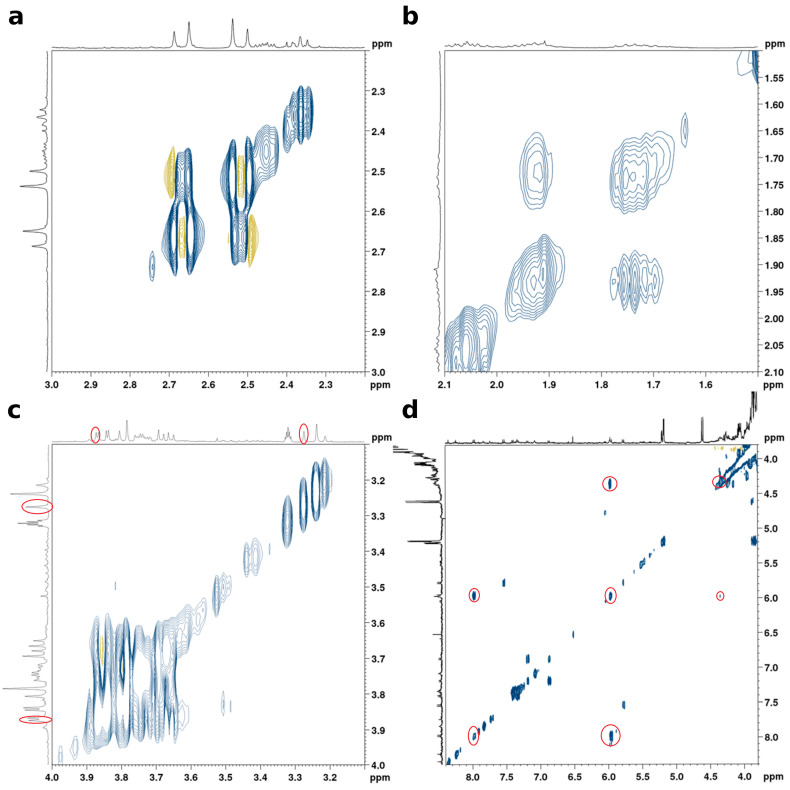
^1^H-^1^H TOCSY spectra showing the spectral regions between 2.20 and 3.00 ppm of *T. magnatum* (**a**), 1.50 and 2.10 ppm of *T. magnatum* (**b**) 3.10 and 4.00 ppm of *T. melanosporum* (**c**) and 8.50 and 3.80 ppm of *T. magnatum* (**d**) assigned to the variables of citric acid, arginine, betaine and UDP-GlcNAc respectively.

For the following relation analysis, the examined variables were reduced to the variables that could clearly be assigned to metabolites by the above-explained procedure. Furthermore, since the highly related variables of neighboring and close-by variables could be assigned to the same signals in the previous section, for clarity, only the respective most important variable was used for the analysis. Figure 5 shows the results of the relation analysis. In addition to four larger clusters, which are discussed in the following section, it is apparent that small groups of variables with very high values for the relation parameter *mean adjusted agreement* (often above 0.9) are built. These relations can be attributed to intramolecular structural relationships and, hence, are assigned to the same metabolite. Specifically, the variables at 2.52–2.53 ppm and 2.68–2.69 ppm, 1.72–1.73 ppm and 1.94–1.95 ppm, 3.27–3.28 ppm and 3.87–3.88 ppm, as well as 5.97–5.98 ppm, 4.34–4.35 ppm, 7.98–7.99 ppm, and 5.51–5.51 ppm, are assigned to citric acid, arginine, betaine, and UDP-GlcNAc, respectively. We confirmed this finding by comparison to the ^1^H-^1^H TOCSY spectra, which are displayed in Figure 6. They show the coupling between the variables of citric acid (Figure 6a), arginine (Figure 6b), and UDP-GlcNAc (Figure 6d). The variables of betaine at 3.27–3.28 and 3.87–3.88 ppm (Figure 6c), however, do not show any coupling since the two signals are not part of the same spin system. The conducted spike-in experiments confirmed the presence of signals from these metabolites in the mentioned spectral regions (Figures S12, S15, S21 and S34). We can therefore conclude that the relationship analyses performed by SMD are consistent with the ^1^H-^1^H TOCSY experiment and are able to reveal chemical structure-based relationships. While ^1^H-^1^H TOCSY reveals chemical correlations within individual spin systems, the example of betaine shows that intramolecular relationships between different spin systems can also be made visible by the application of SMD. 

The assignment of various variables to the same metabolite based on the SMD relation analysis is largely in agreement with the results of correlation analysis, which is usually applied for this purpose in STOCSY experiments (see Figure S4). However, the mean adjusted agreement values of variables of the same metabolite differ much more from those of different metabolites, which simplifies the assignment considerably.

Since the signals from multiple metabolites can be superimposed in individual buckets, it can be difficult to determine which molecules provide the relevant information for classification when only variable selection is performed. SMD relation analysis, however, can be applied to analyze these buckets in more detail: the variable at 3.23–3.24 ppm, for example, was associated with choline-O-sulfate, glycerophosphorylcholine (GPC), and arginine. While this variable shows high values of the relation parameter for another selected variable assigned to choline-O-sulfate at 4.49–4.50 ppm, the additional variables associated with GPC or arginine are characterized by relation values around zero. We can therefore assume that the classification-relevant information contained in the variable at 3.23–3.24 ppm originates from choline-O-sulfate. In contrast, the variables at 3.82–3.83 ppm and 3.41–3.42 ppm, which were assigned to trehalose and ribonate, and trehalose and proline, respectively, show relationships with both other variables assigned to trehalose, e.g., at 5.18–5.19 ppm, and variables at 4.13–4.14 ppm and 4.08–4.09 ppm assigned to ribonate and proline, respectively. Thus, in both cases, both metabolites are relevant for the classification. In summary, the parameter mean adjusted agreement for the analysis of variable relationships is a useful additional element to complement the toolbox for the identification of metabolites in authentication experiments.

#### 3.4.3. Relations of Variables from Different Metabolites

**Figure 7:**
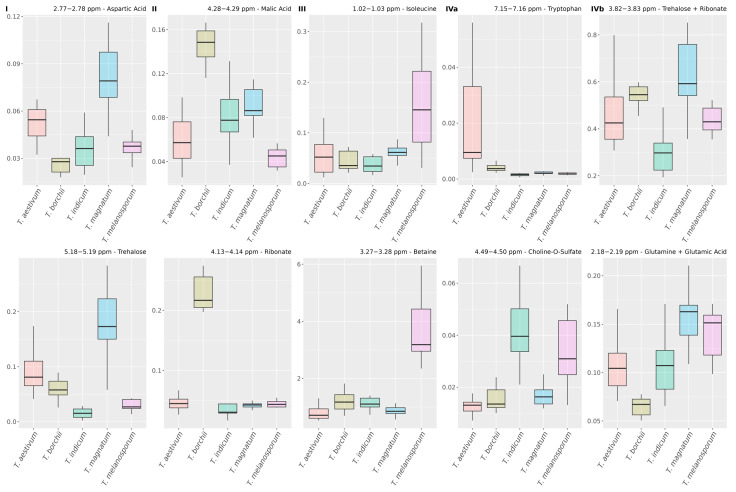
Boxplots of two representative variables for each cluster in Figure 5. The boxplots of the respective other variables of the clusters are shown in Figures S4–S8.

In Figure 5, four clusters are built based on the mutual information the respective metabolites contribute for classification. This information can be examined in more detail in Figure 7, in which boxplots of exemplary variables of each cluster are displayed, and in Figures S4–S8, showing boxplots of all variables contained in the respective clusters. 

Cluster I contains various variables with high intensities for *T. magnatum* (Figure 7I and Figure S5). The high values for the *mean adjusted agreement* of UDP-GlcNAc and trehalose could be explained by the biosynthesis of chitin, in which both molecules are involved in [50], indicating a different cell wall composition of *T. magnatum*. The relations between signals from arginine, proline, and lysine could be explained by structural similarities because they are all amino acids with nitrogenous side chains. Since these variables also show strong relations to asparagine and aspartic acid, which are important nitrogen carriers in plants [51,52], this could indicate differences in amino acid metabolism, nitrogen assimilation, and growth of *T. magnatum.*

Cluster II contains variables with specific classification information for *T. borchii* (see Figure 7II and Figure S6). The variables assigned to malic and fumaric acid show very high values for the relation parameter, thus building a small subcluster. Since fumaric acid is converted to malic acid in the tricarboxylic acid cycle (TCA), this could indicate principal differences in the energy metabolism of *T. borchii.* In the fungus *Rhizopus arrhizus*, the accumulation of malic and fumaric acid could be traced back to the TCA and glyoxylic acid pathway, which could also be the source of the enrichment in *T. borchii* [53,54]. However, the specific difference of *T. borchii* is not apparent from all selected variables of the TCA, and variables that are associated with citric acid are grouped in cluster I, providing vastly different information for the classification model (see Figures S5 and S6). This could be explained by the fact that citric acid acts as an intermediate, while both fumaric and malic acid act as main products. A variable at 4.13–4.14 ppm assigned to ribonate is also grouped in Cluster II. This is in accordance with our previous study because this metabolite, which is also related to energy metabolism, was identified as an exclusive marker for *T. borchii* [38]. In our analysis, it becomes apparent that high concentrations of ribonate are highly related to low concentrations of histidine in *T. borchii*. This could be explained by the presence of *Pseudomonas*, which are known to populate *T. borchii* [55], because they use histidine as a carbon source [56]. In summary, the metabolites of Cluster II show differences in the energy metabolism of *T. borchii*, which can be used to uniquely identify this species. 

Cluster III is specific for the identification of *T. melanosporum* and contains five variables with comparatively high concentrations for this species (Figure 7III and Figure S7). Two of these variables were assigned to betaine and the other three to isoleucine, leucine, and valine. The high values of the relationship parameter for the latter three can be explained by the fact that these metabolites are structurally and functionally very similar amino acids, called branched chain amino acids (BCAAs). Since they show specific classification information for *T. melanosporum*, differences in the synthesis and usage of BCAAs, which are well studied for fungi, can be assumed [57]. Betaine is known to be built in plants as a widespread response against environmental stress [58]. Hence, *T. melanosporum* could have a different stress tolerance or react differently to it than the other analyzed species. 

Cluster IV contains variables with inhomogeneous classification information and we split them into two subclusters. Cluster IVa, the first subcluster (Figure 7IVa and Figure S8), contains a variable at 7.15–7.16 ppm that has a very high concentration for *T. aestivum* and thus provides very specific classification information for this species. In *Cryptococcus neoformans*, tryptophan uptake and biosynthesis is essential for the survival of the organism at lower temperatures or when non-preferred nitrogen sources are available [59]. Higher tryptophan concentrations in *T. aestivum* could indicate that this species reacts differently to such external influences than the other species. The variables assigned to choline-O-sulfate show specific classification information to separate *T. indicum* and *T. melanosporum* from the other truffle species. Since it has been shown that fungi use this metabolite as a source of sulfur, this could demonstrate that the *Tuber* species have different sulfur metabolism [60]. 

Cluster IVb contains four variables (Figure 7IVb and Figure S9). Two of them, which are assigned to glutamic acid and glutamine, are specific for the identification of *T. borchii* with very low levels for this class. They are therefore related to Cluster II, confirming the conclusion that this species could differ in energy metabolism. The variable at 3.82–3.83 ppm provides specific information for the classification of *T. indicum* and is assigned to ribonate and trehalose. The comparison of the classification of truffle species based on variables containing only ribonate or trehalose (see Figure 7I,II) shows that this bucket is indeed characterized by an overlap of the contributions of both metabolites. This is confirmed by the strong relations to the other variables of these metabolites, which were also discussed previously (see Section 3.4.2). However, since the increased concentration of *T. melanosporum* is not caused by one of the two metabolites, a third, unfortunately unidentified metabolite probably influences the variable at 3.82–3.83 ppm. The variable at 4.22–4.23 ppm associated with threonine shows unique classification information for *T. aestivum*. It is therefore strongly related to the other variable contributing this information at 7.15–7.16 ppm, which is assigned to tryptophan and was discussed in the previous paragraph. Threonine has been identified as a common residue from dephosphorylation reactions of proteins within *Saccharomyces cerevisiae* and other fungi, suggesting a different protein metabolism of *T. aestivum* [61]. 

In summary, the relationship analysis with SMD identified groups of variables with similar classification information that can be used to interpret class differences. Since these relationships are not apparent in the correlation analysis (see Figure S4), our analysis shows the benefit of including classification information in the relationship analysis of variables from NMR data. 

## 4. Conclusions

In this study, using the classification of different truffle species, we demonstrate that the random forest black box for ^1^H NMR metabolomics data can be opened by the application of SMD. We show this by the selection of important variables and the comprehensive analysis of variable relations based on their mutual impact on the random forest model. Groups of metabolites characteristic of specific species could be identified and linked to meaningful biological relationships. In addition, based on the SMD relation parameter, variables assigned to the same signals and metabolites could be identified and buckets with superimposed information could be unraveled. In summary, this analysis shows the potential of SMD for the comprehensive analysis of complex ^1^H NMR metabolomics data to select and characterize the variables involved and support the identification and interpretation of the corresponding metabolites. 

## Figures and Tables

**Table 1:** Overview of the truffle samples used in this study.

	*T. aestivum*	*T. borchii*	*T. indicum*	*T. magnatum*	*T. melanosporum*
Amount	28	7	12	21	12
Color	black	white	black	white	black

**Table 2:** Parameters used for RF-based approaches with p representing the total number of variables.

Approach	Parameter	Description	Value
RF	ntree	number of trees	10,000
	min.node.size	number of samples in terminal node	1
	mtry	number of candidate variables	157 (p^3/4^) ^1^
	case. weights	weights for sampling of training observations	chosen according to the size of the respective class
SMD	s	Predefined number of surrogate splits	42 (p ∙ 0.05)
Boruta	pValue	applied importance measure	impurity_corrected
	importance	confidence level	0.01
	maxRuns	maximum number of importance source runs	157 (p^3/4^) ^1^

**^1^** Motivated by [32].

**Table 3:** Result of the random forest classification of truffle samples. An out-of-bag error of 0% corresponding with a classification accuracy of 100% was obtained.

	*T. aestivum*	*T. borchii*	*T. indicum*	*T. magnatum*	*T. melanosporum*	Sensitivity [%]
***T. aestivum***	28	0	0	0	0	100
***T. borchii***	0	7	0	0	0	100
***T. indicum***	0	0	12	0	0	100
***T. magnatum***	0	0	0	21	0	100
***T. melanosporum***	0	0	0	0	12	100
**Specificity [%]**	100	100	100	100	100	

## Data Availability

Data are provided in the supplement.

## Supplementary Materials

The following supporting information can be downloaded at: https://www.mdpi.com/article/10.3390/metabo13101075/s1, Figure S1: Results of the principal component analysis; Figure S2: Relations of variables selected by SMD; Figure S3: Relations of variables selected by Boruta; Figure S4: Correlations of variables selected by SMD; Figures S5–S9: Boxplots of selected variables; Figures S10–S34: Results of spike-in experiments; Table S1: Data used for the RF analyses; Table S2: List of the variables selected by SMD and Boruta.
