# Supplementary material for: Opening the Random Forest Black Box of 1H NMR Metabolomics Data by the Exploitation of Surrogate Variables
Source: Metabolites. 2023 Oct 13;13(10):1075. doi: 10.3390/metabo13101075 (PMC10608983; doi:10.3390/metabo13101075)
Supplement: Supplementary file 1 [file metabolites-13-01075-s001.zip › Supplementary_figures.pdf]

Supplementary Figures

Opening the Random Forest Black Box of ^{1}H NMR Metabolomics Data by the Exploitation of Surrogate Variables

Soeren Wenck [1,†], Thorsten Mix [2,†], Markus Fischer [1], Thomas Hackl [1,2] and Stephan Seifert [1,*]

[1] Institute of Food Chemistry, Hamburg School of Food Science, University of Hamburg, Grindelallee 117, 20146 Hamburg, Germany; markus.fischer@uni-hamburg.de (M.F.); thomas.hackl@uni-hamburg.de (T.H.)
[2] Institute of Organic Chemistry, University of Hamburg, Martin-Luther-King-Platz 6, 20146 Hamburg, Germany; thorsten.mix@uni-hamburg.de
* Correspondence: stephan.seifert@uni-hamburg.de; Tel.: +49-40-42838-8818
† These authors contributed equally to this work.

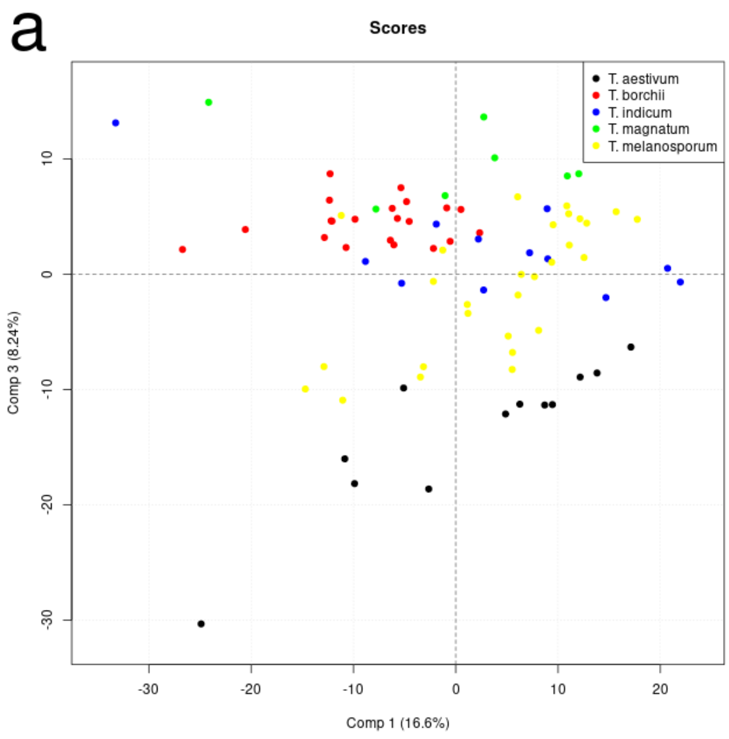

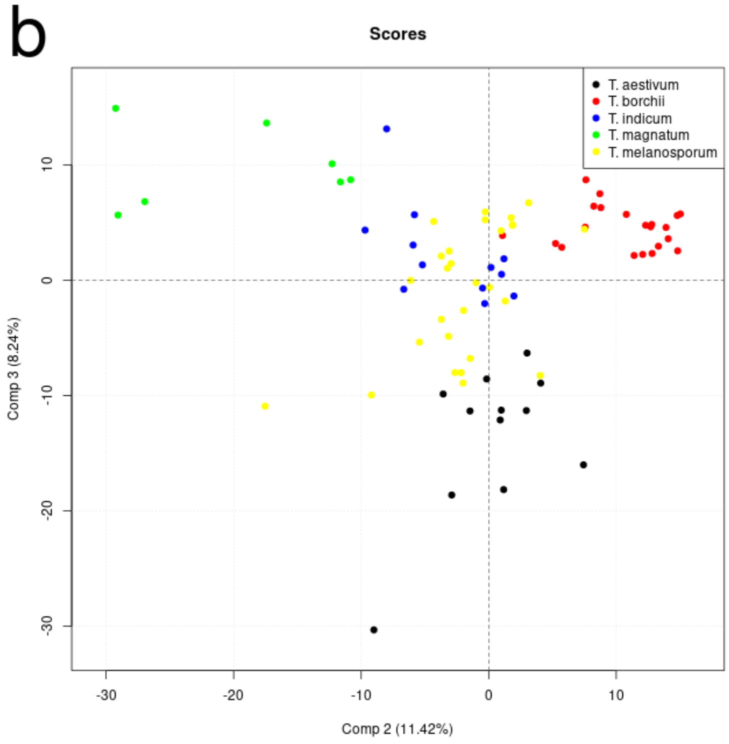

Figure S1: Results of the principal component analysis of *Tuber* samples: Scores of the first and third (a) and second and third (b) principal components are shown.

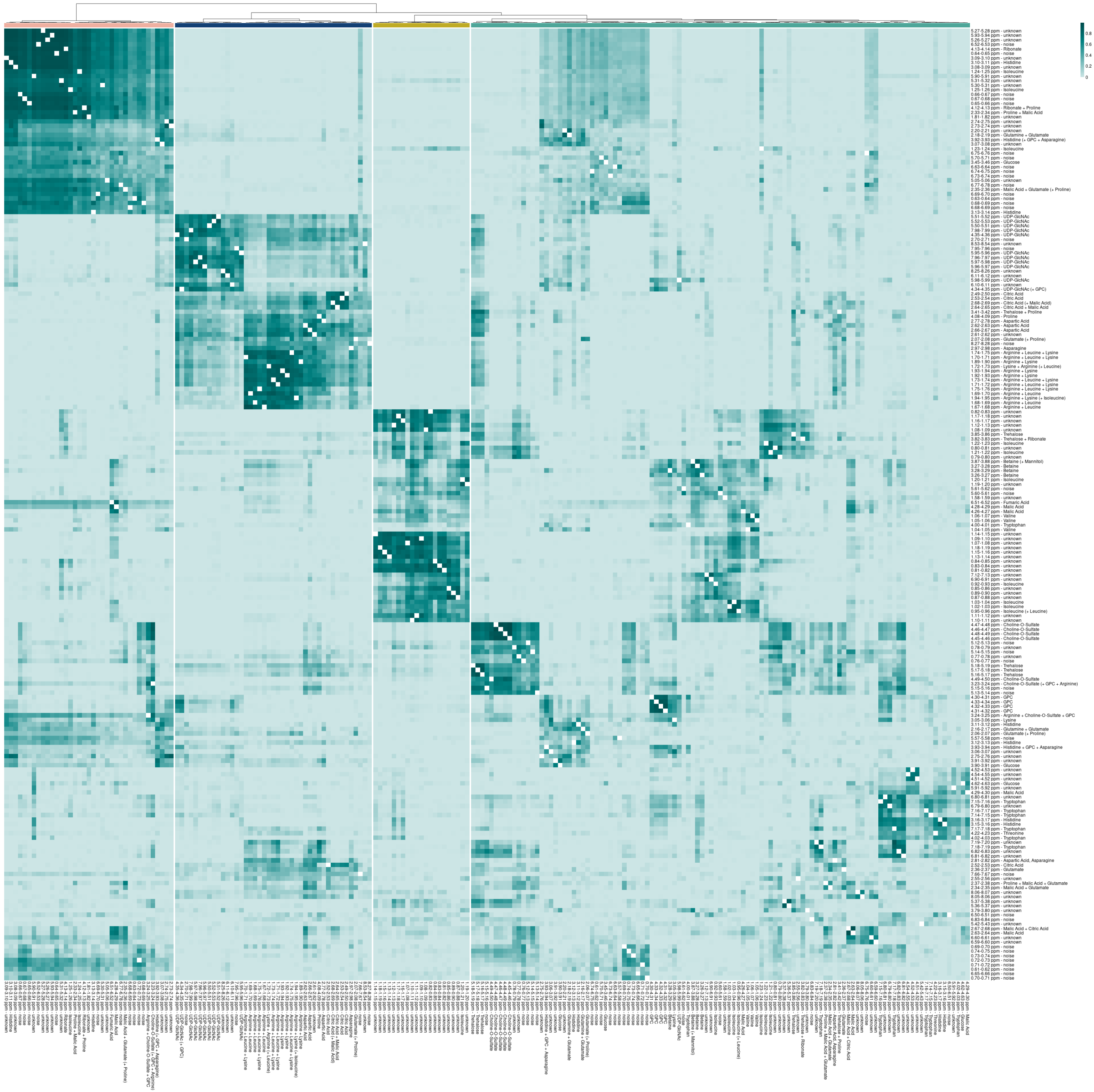

Figure S2: Results of the variable relation analysis based on the mean adjusted agreement of variables selected by SMD. For the hierarchical cluster analysis, Euclidean distances and Ward´s algorithm were applied

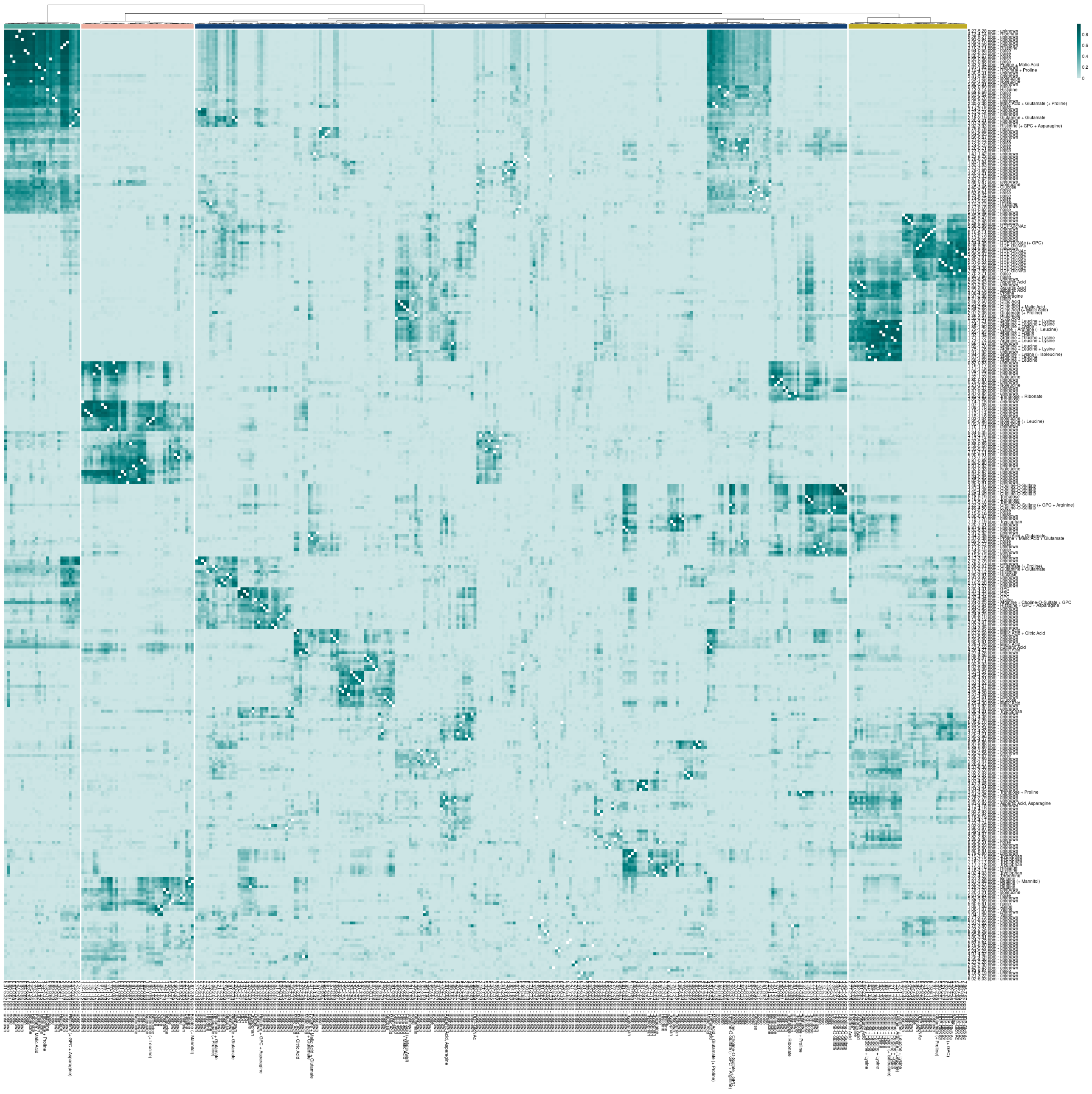

Figure S3: Results of the variable relation analysis based on the mean adjusted agreement of variables selected by Boruta. For the hierarchical cluster analysis, Euclidean distances and Ward´s algorithm were applied.

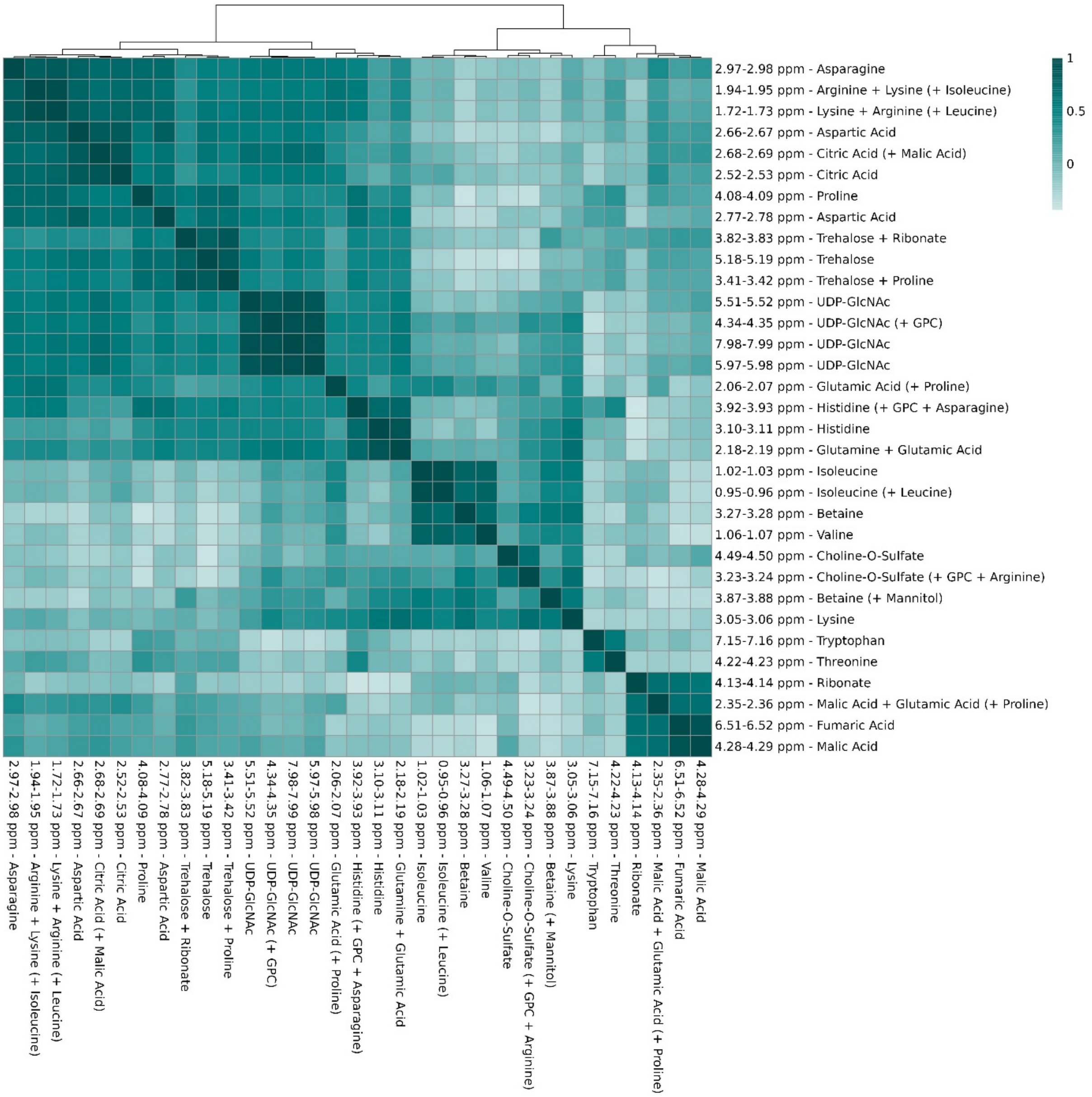

Figure S4: Results of the relation analysis based on pairwise Pearson correlation coefficients of the variables selected by SMD. For the hierarchical cluster analysis, Euclidean distances and Ward´s algorithm were applied.

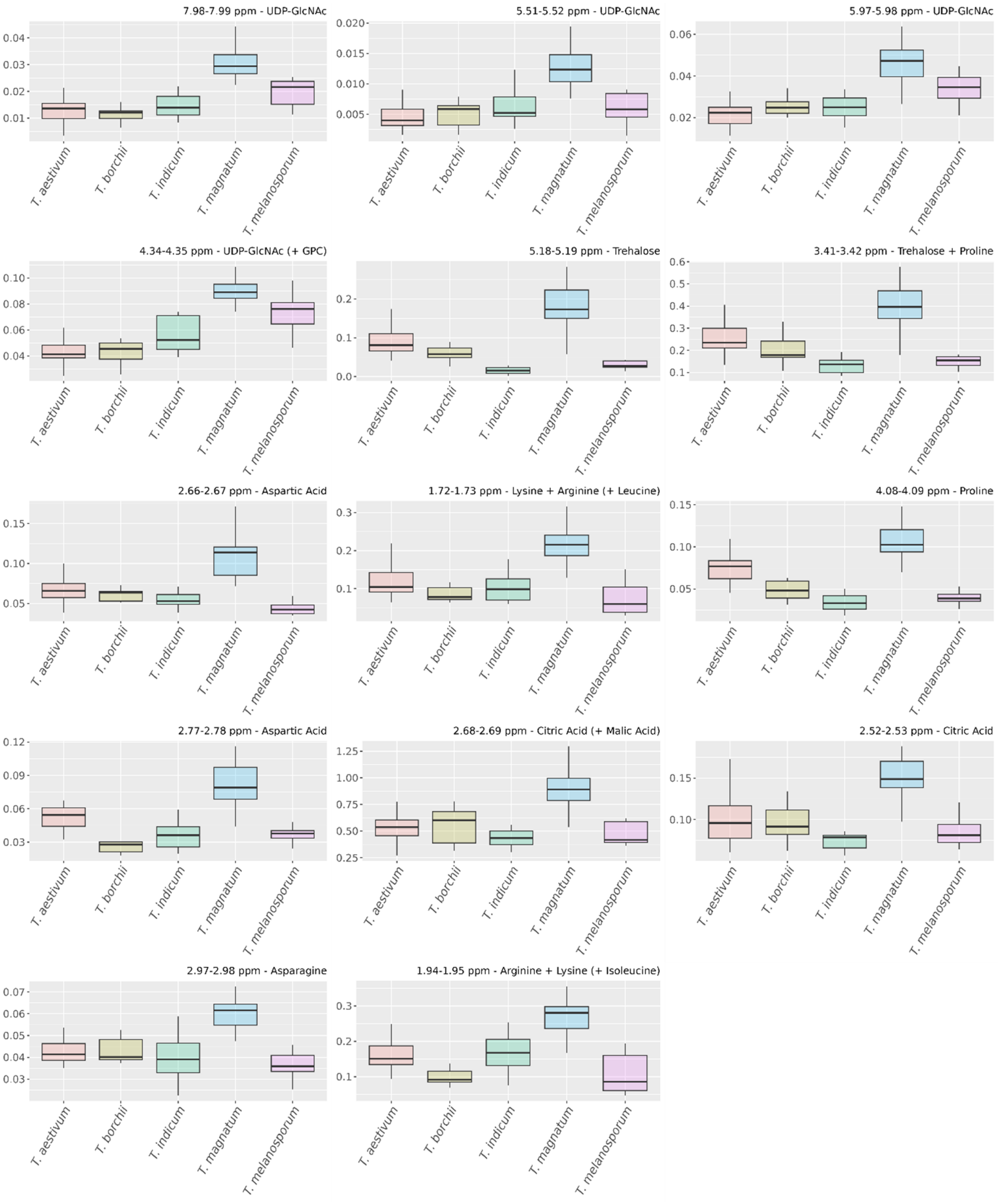

Figure S5: Boxplots of the selected variables in cluster I of Figure 4.

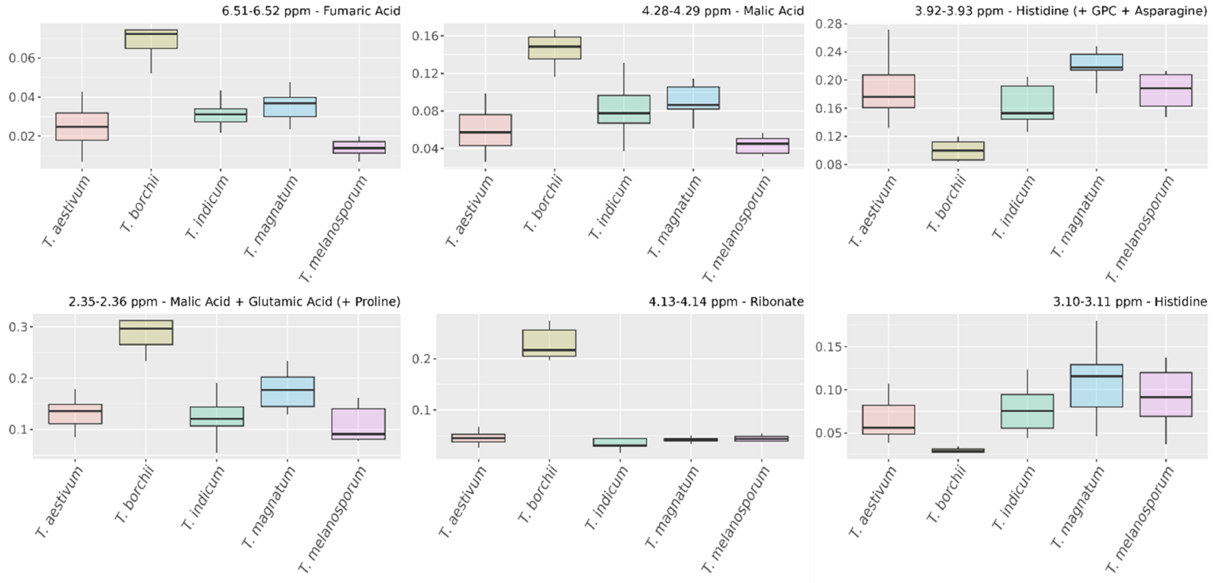

Figure S6: Boxplots of the selected variables in cluster II of Figure 4.

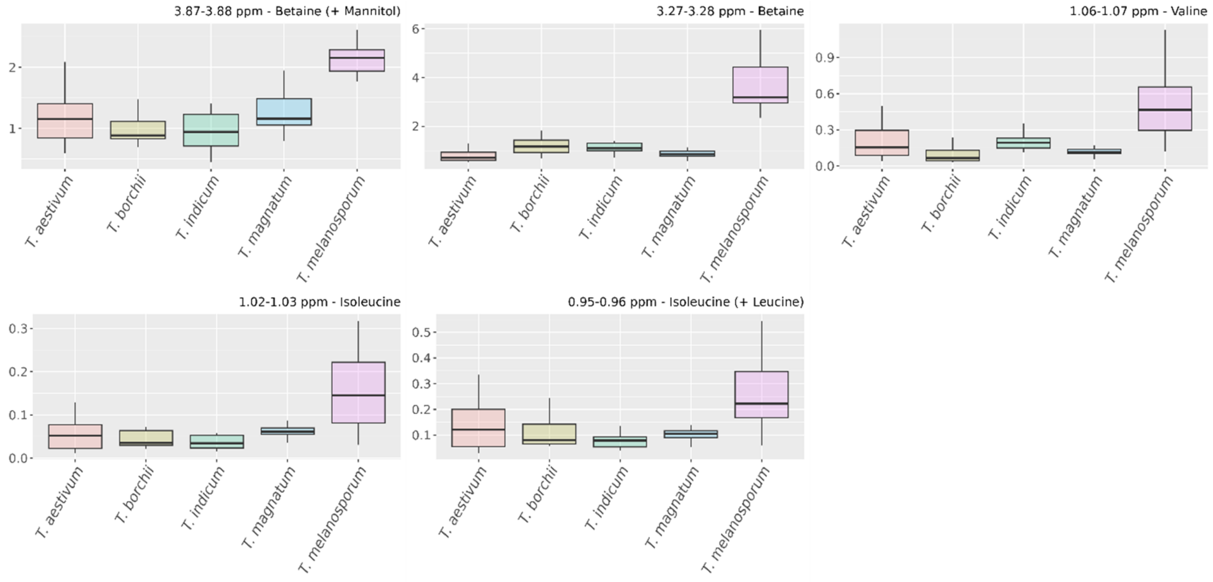

Figure S7: Boxplots of the selected variables in cluster III of Figure 4.

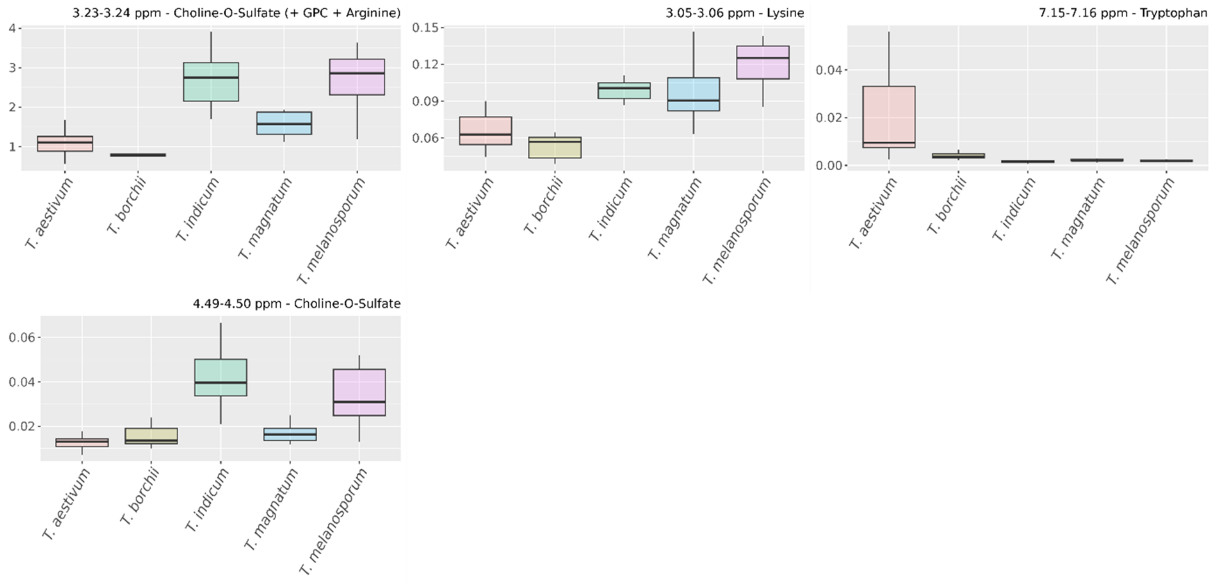

Figure S8: Boxplots of the selected variables in cluster IVa of Figure 4.

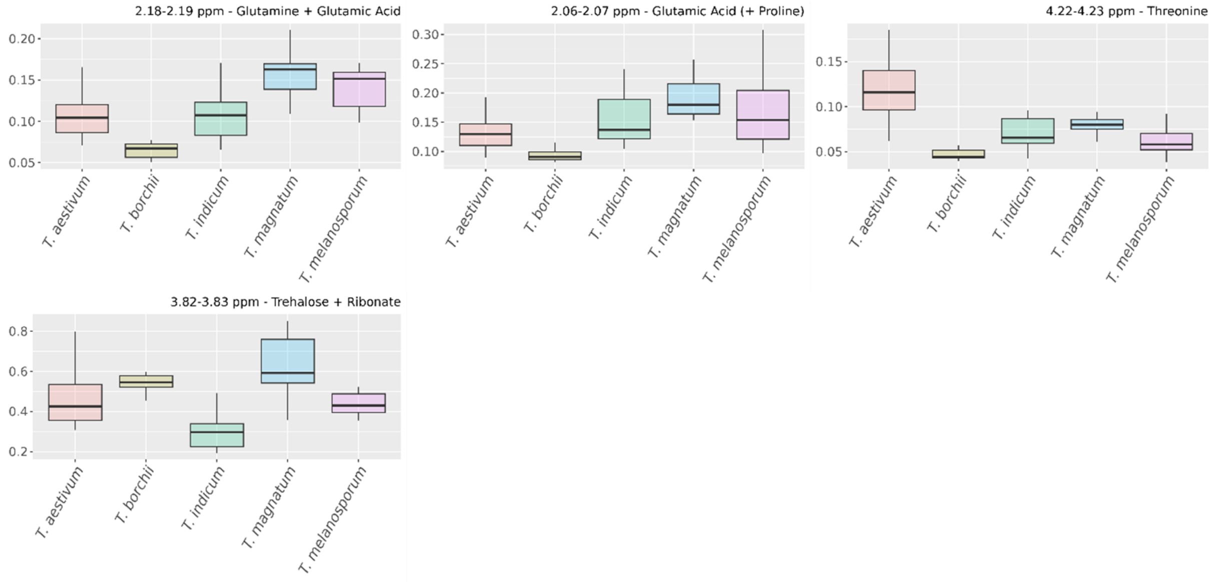

Figure S9: Boxplots of the selected variables in cluster IVb of Figure 4.

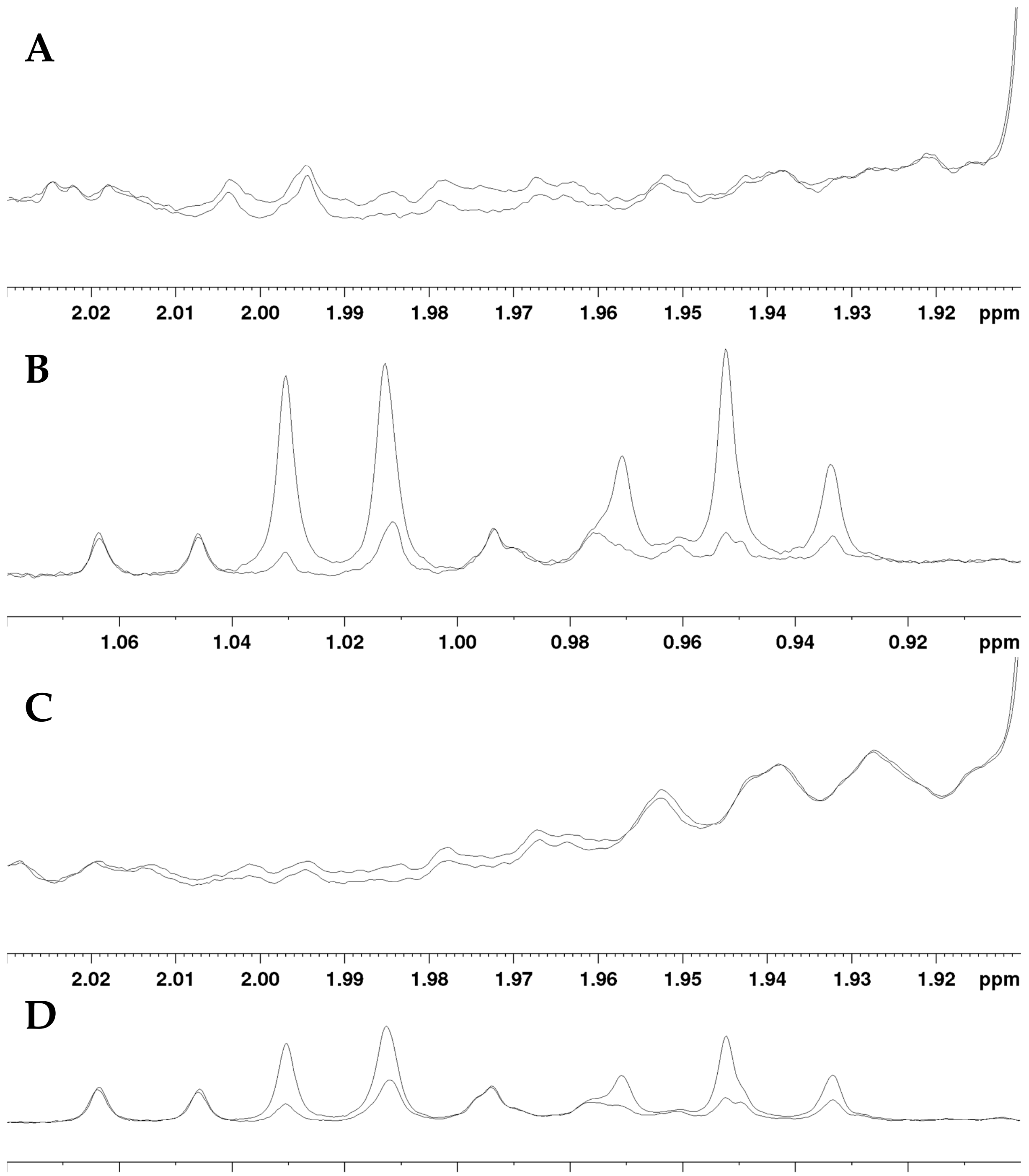

Figure S10: Results of spike-in experiment with 40 µg L-Isoleucine at 400 MHz. A: 2.04-1.90 ppm in *T. aestivum,* B: 1.08-0.90 ppm in *T. aestivum,* C: 2.04-1.90 ppm in *T. magnatum,* D: 1.08-0.90 ppm in *T. magnatum*.

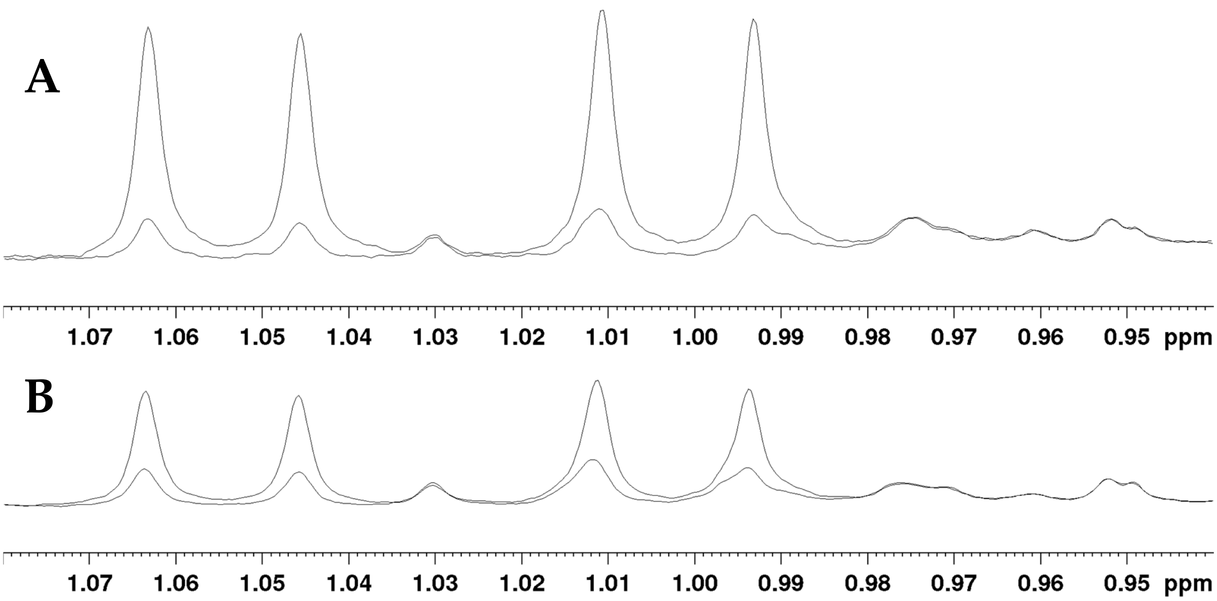

Figure S11: Results of spike-in experiment with 40 μg L-Valine at 1.08-0.94 ppm at 400 MHz. A: *T. aestivum*, B: *T. magnatum*

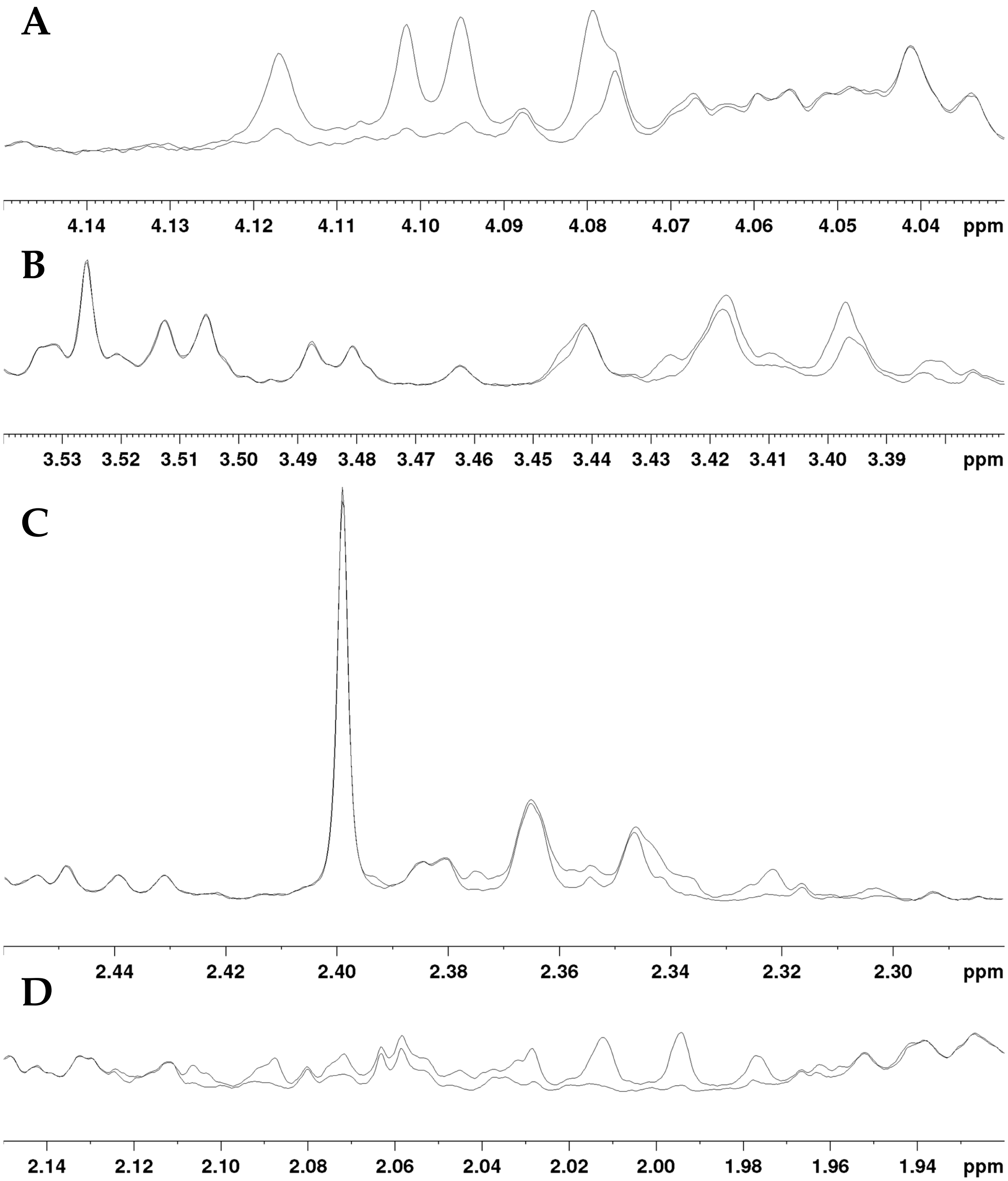

Figure S13: Results of spike-in experiment with 100 µg L-Prolin at 400 MHz in *T. magnatum.* A: 4.45-4.03 ppm, B: 3.54-3.37 ppm, C: 2.46-2.28 ppm, D: 2.15-1.92 ppm

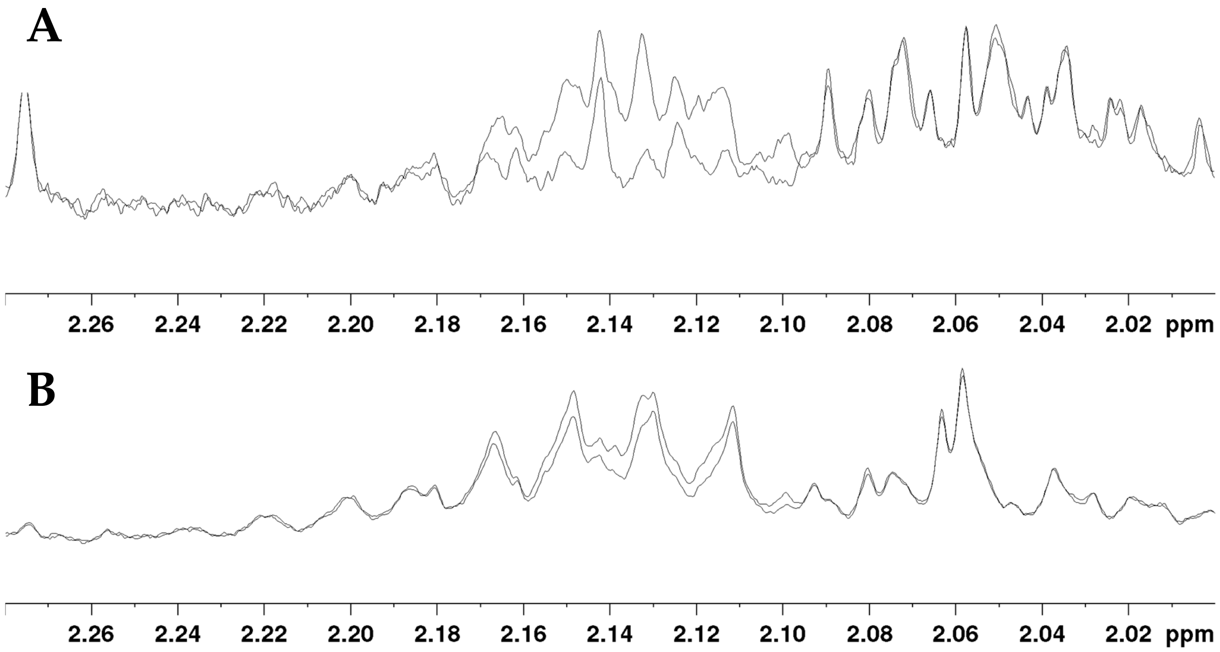

Figure S14: Results of spike-in experiment with 40 µg L-Glutamine at 2.28-2.00 ppm at 400 MHz. A: *T. aestivum*; B: *T. magnatum*.

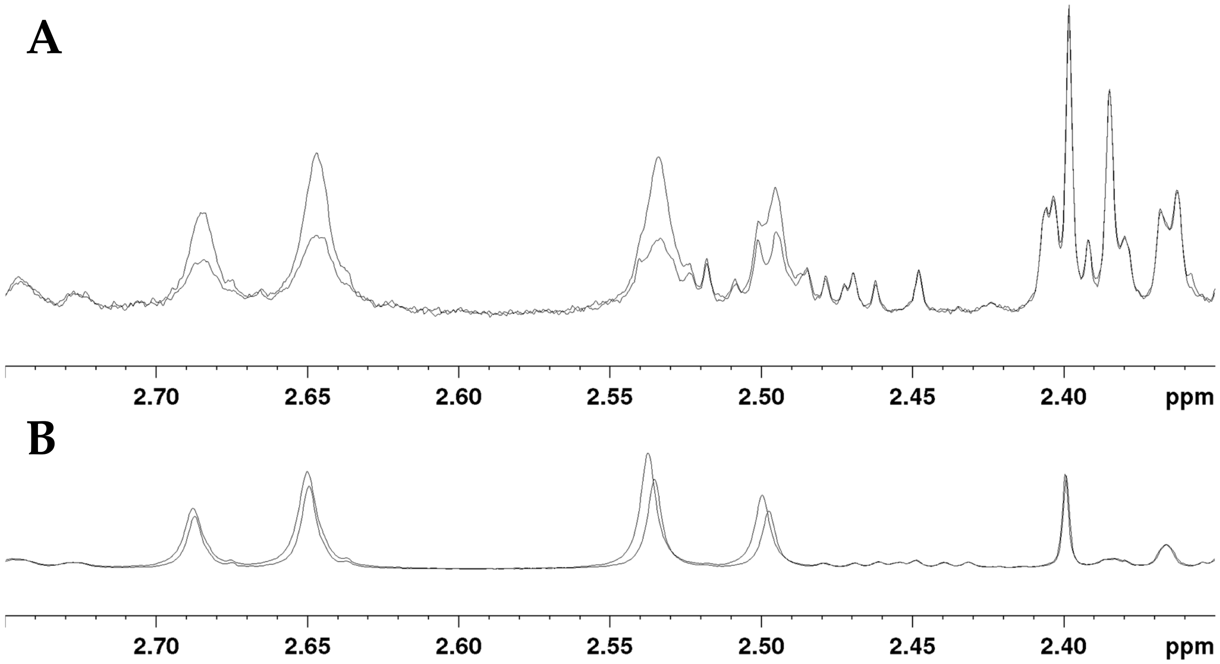

Figure S15: Results of spike-in experiment with L-Citric Acid at 2.75-2.35 ppm at 400 MHz. A: 40 µg in *T. aestivum*, B: 200 µg in *T. magnatum*

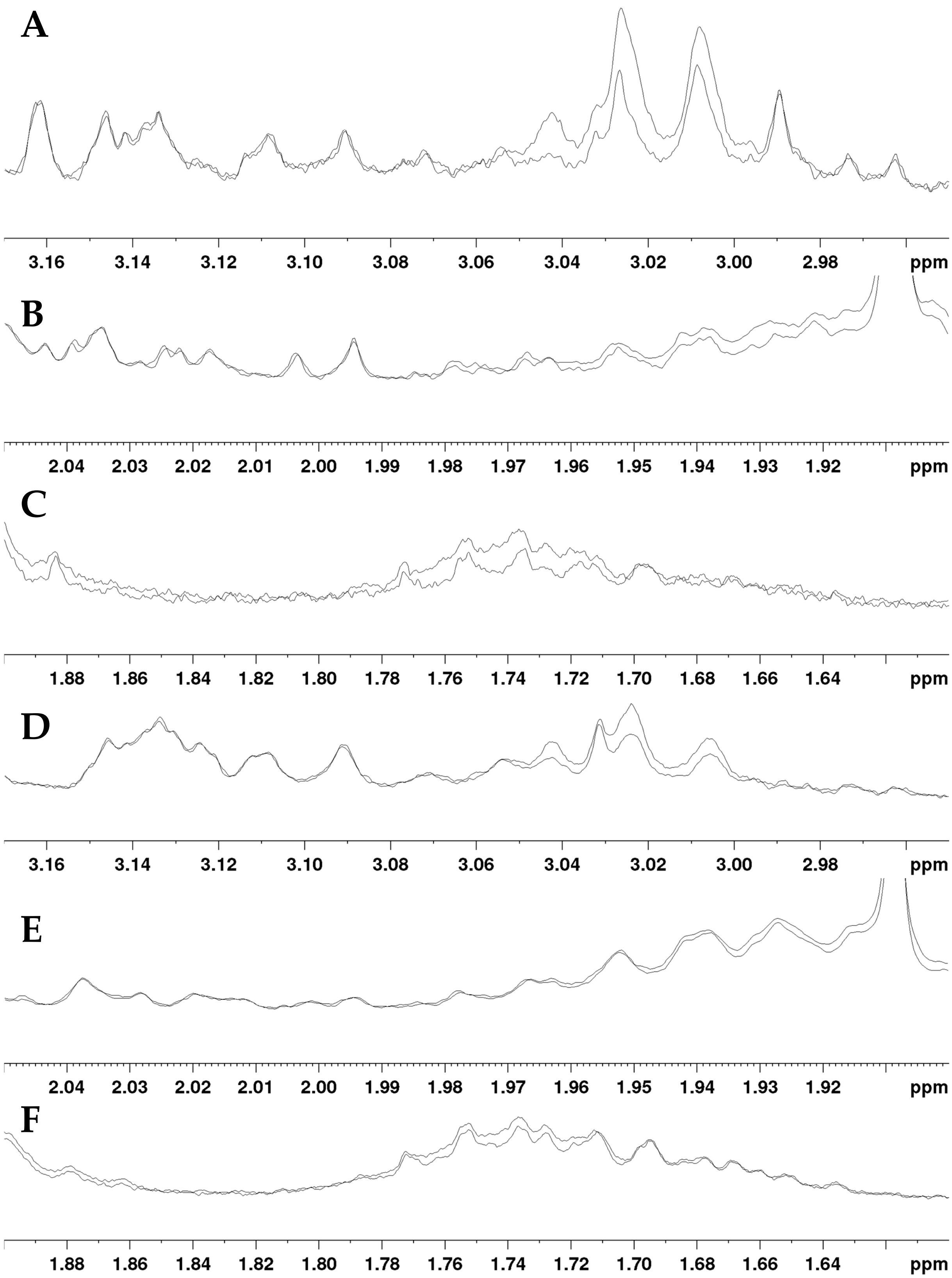

Figure S16: Results of spike-in experiment at 400 MHz.with 40 µg L-Lysine. A: 3.17-2.96 ppm in *T. aestivum,* B: 2.05-1.90 ppm in *T. aestivum*, C: 1.90-1.60 ppm in *T. aestivum,* D: 3.17-2.96 ppm in *T. magnatum,* E: 2.05-1.90 ppm in *T. magnatum*, F: 1.90-1.60 ppm in *T. magnatum.*

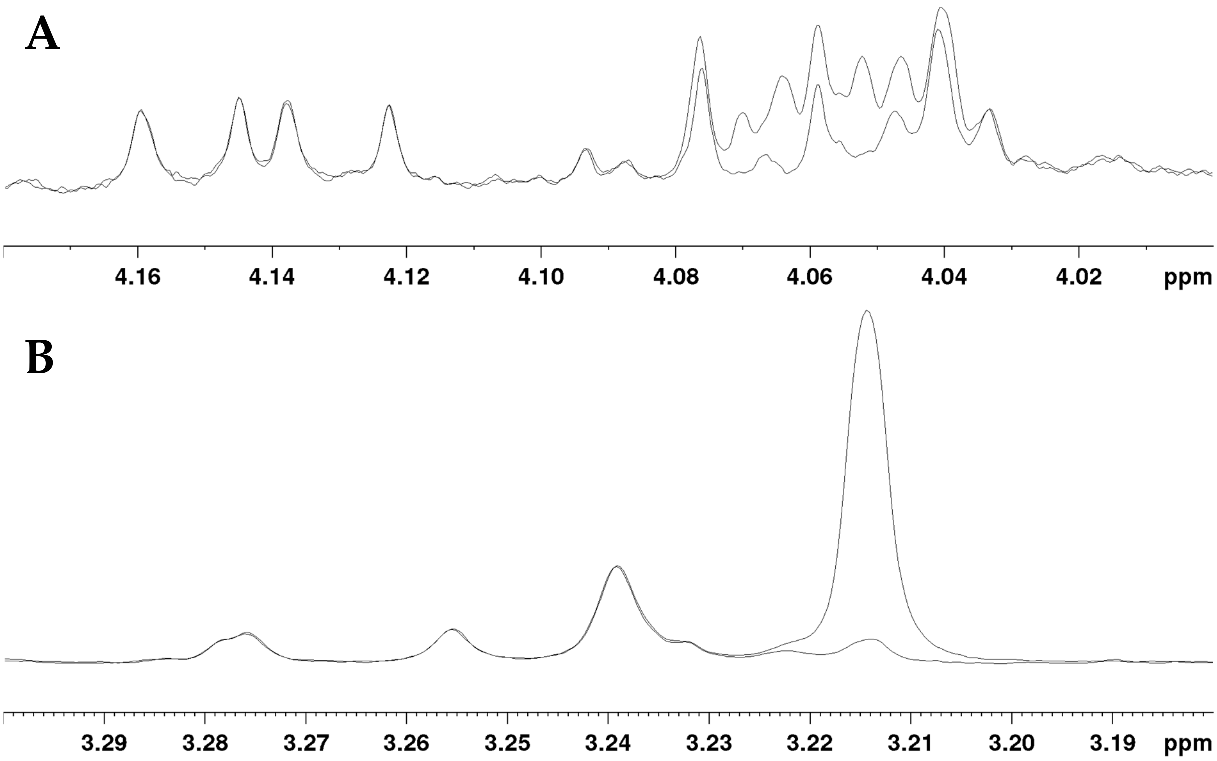

Figure S17: Results of spike-in experiment with 40 μg Choline at 400 MHz in *T. aestivum.* A: 4.18-4.00 ppm, B: 3.30-3.18 ppm

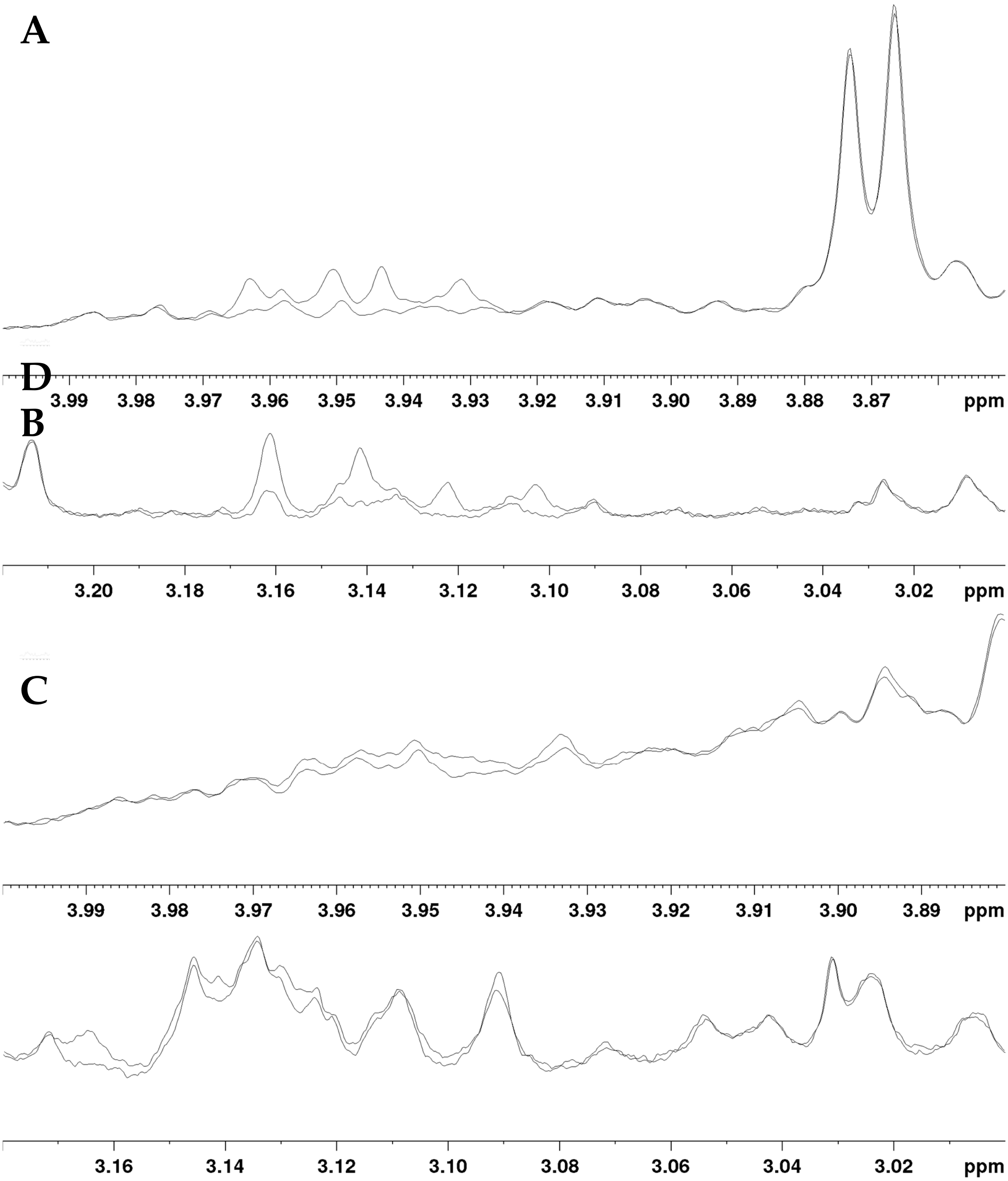

Figure S18: Results of spike-in experiment L-Histidine at 400 MHz. A: 4.00-3.85 ppm with 100 µg in *T. aestivum*, B: 3.22-3.00 ppm with 100 µg in *T. aestivum*, C: 4.00-3.88 ppm with 40 µg in *T. magnatum* at, D: 3.18-3.00 ppm with 40 µg in *T. magnatum*.

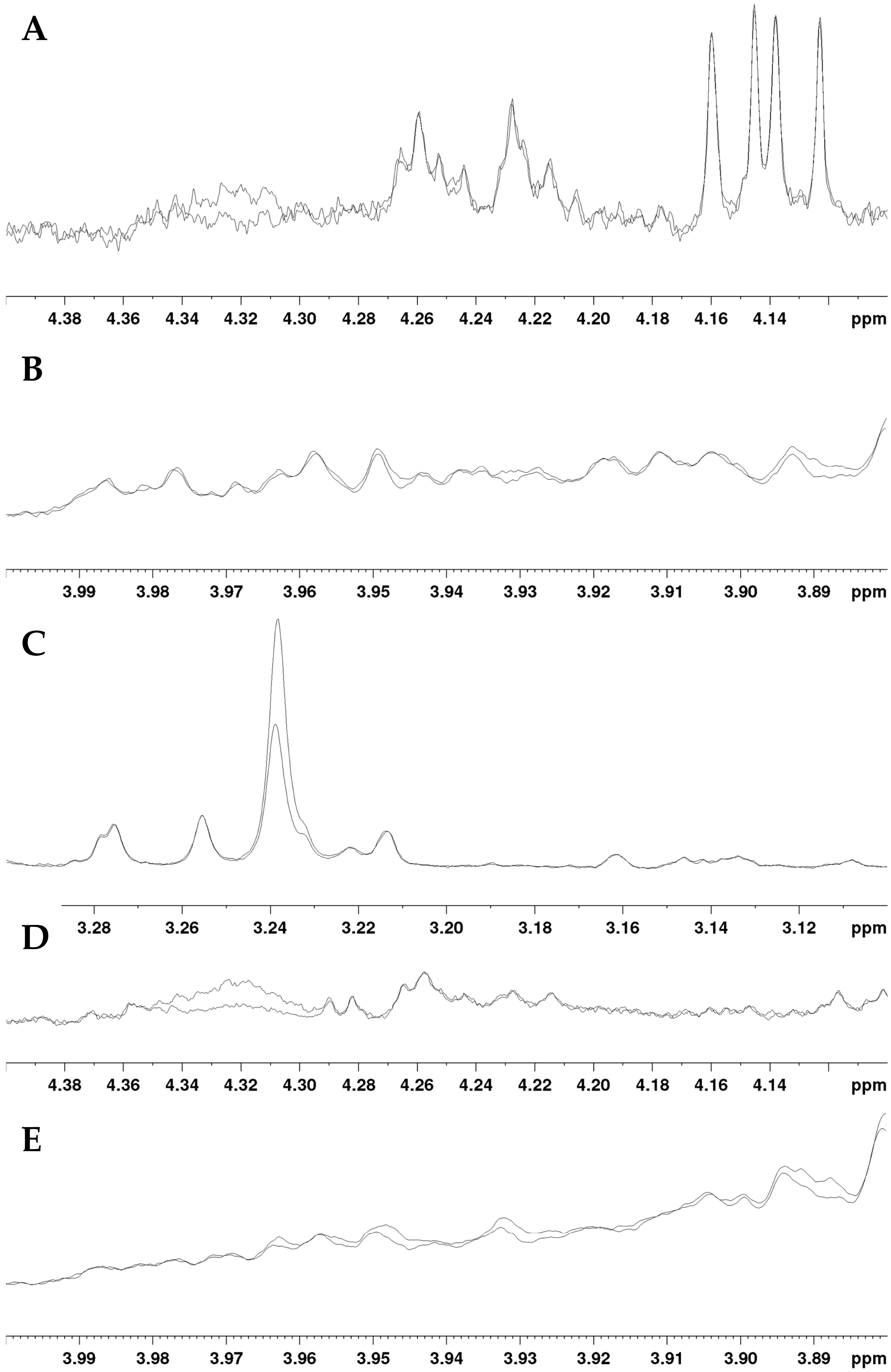
A
4.38 4.36 4.34 4.32 4.30 4.28 4.26 4.24 4.22 4.20 4.18 4.16 4.14 ppm
B
3.99 3.98 3.97 3.96 3.95 3.94 3.93 3.92 3.91 3.90 3.89 ppm
C
3.28 3.26 3.24 3.22 3.20 3.18 3.16 3.14 3.12 ppm
D
4.38 4.36 4.34 4.32 4.30 4.28 4.26 4.24 4.22 4.20 4.18 4.16 4.14 ppm
E
3.99 3.98 3.97 3.96 3.95 3.94 3.93 3.92 3.91 3.90 3.89 ppm

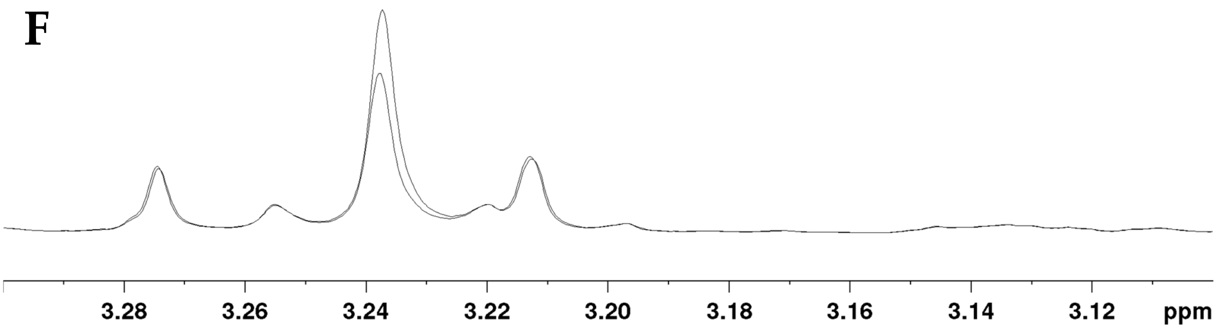

Figure S19: Results of spike-in experiment with Glycerophosphorylcholine (GPC) at 400 MHz. A: 4.40-4.10 ppm with 20 µg in *T. aestivum*, B: 4.00-3.88 ppm with 20 µg in *T. aestivum*, C: 3.30-3.10 ppm with 20 µg in *T. aestivum*, D: 4.40-4.10 ppm with 50 µg in *T. magnatum*, E: 4.00-3.88 ppm with 50 µg in *T. magnatum*, F: 3.30-3.10 ppm with 50 µg in *T. magnatum*.

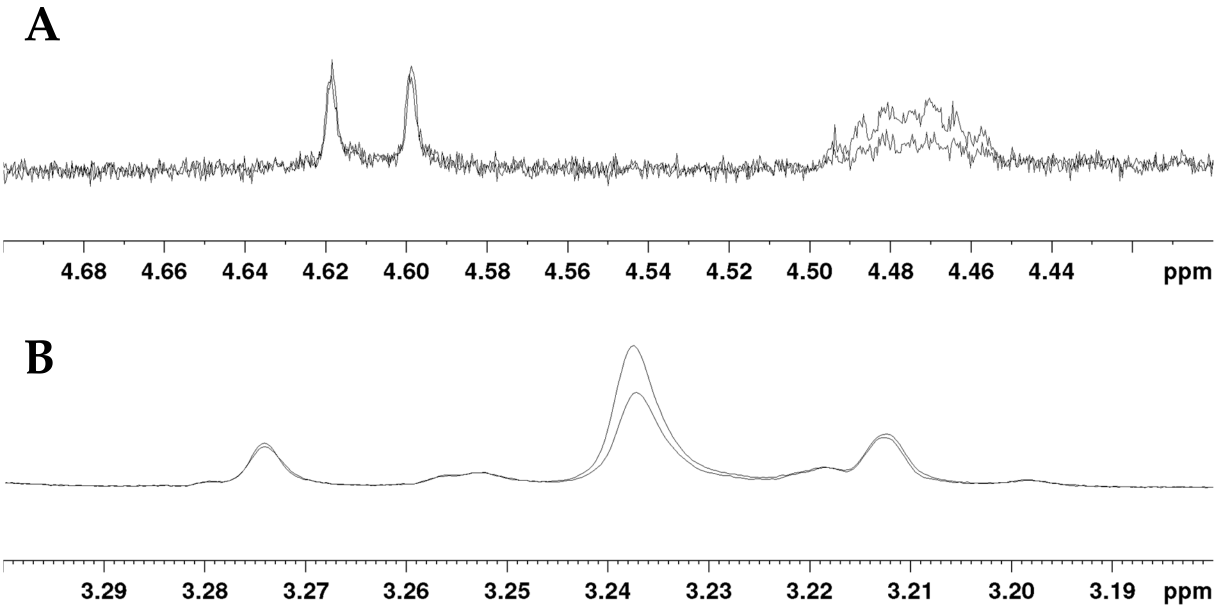

Figure S20: Results of spike-in experiment with 100 µg Choline-O-Sulfate in *T. magnatum* at 400 MHz. A: 4.70-4.40 ppm, B: 3.30-3.18 ppm.

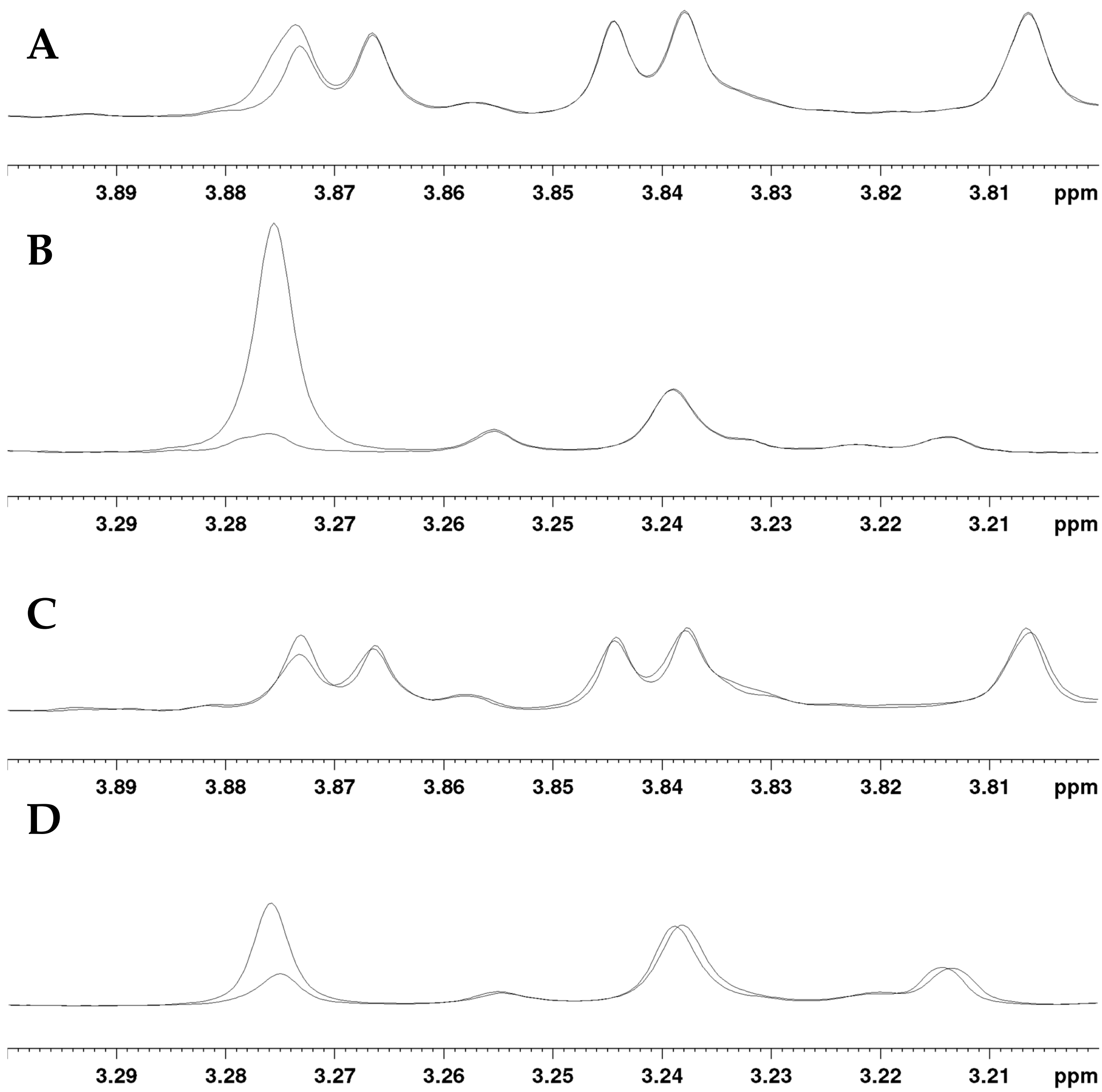

Figure S21: Results of spike-in experiment with 40 µg Betaine at 400 MHz. A: 3.90-3.80 ppm in *T. aestivum,* B: 3.30-3.20 ppm in *T. aestivum,* C: 3.90-3.80 ppm in *T. magnatum*, D: 3.30-3.20 ppm in *T. magnatum*.

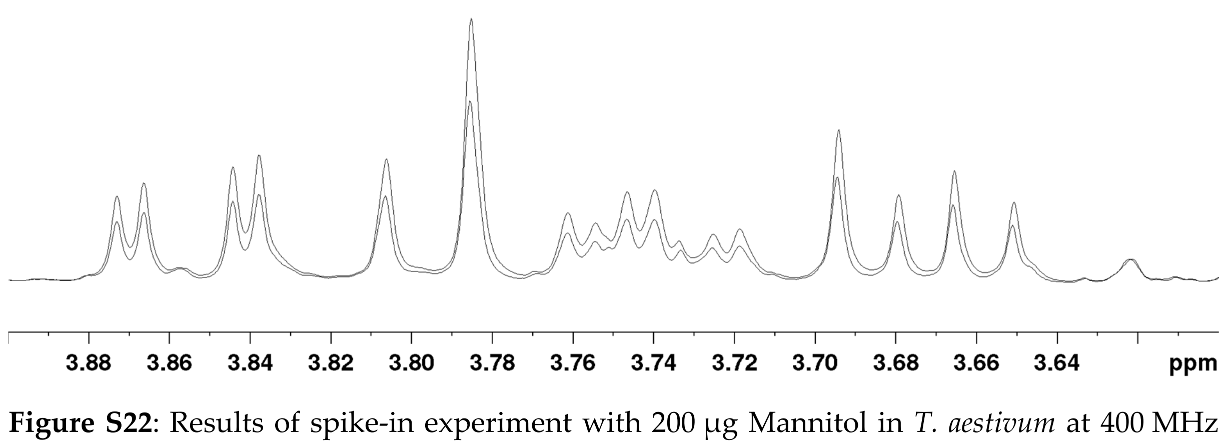

Figure S22: Results of spike-in experiment with 200 µg Mannitol in *T. aestivum* at 400 MHz between 3.90-3.60 ppm

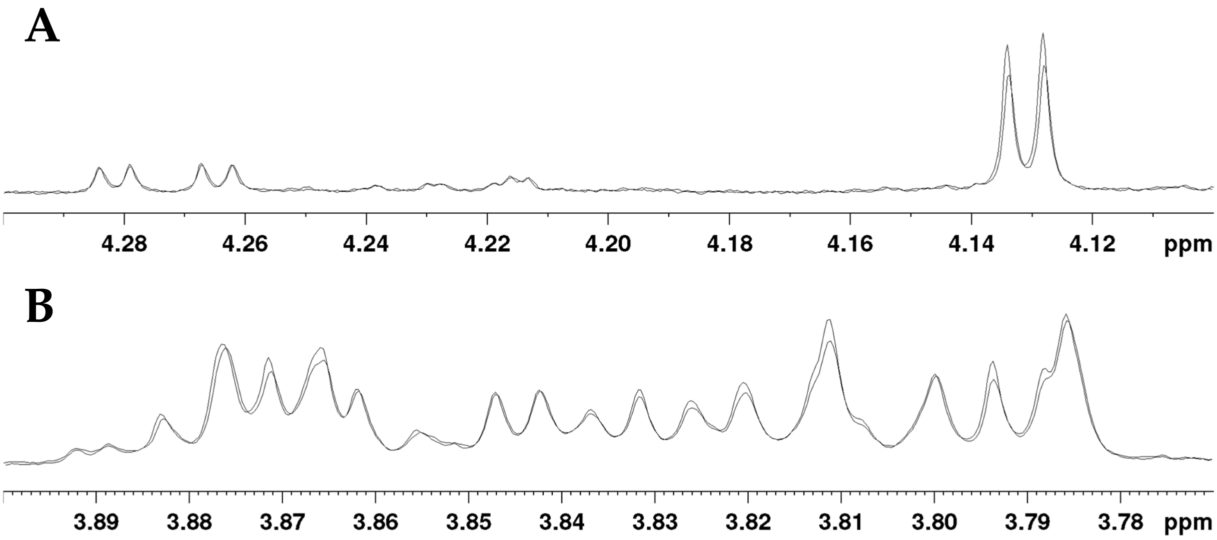

Figure S23: Results of spike-in experiment with 30 μg Ribonate in *T. borchii* at 600 MHz. A: 4.30-4.10 ppm, B: 3.90-3.77 ppm

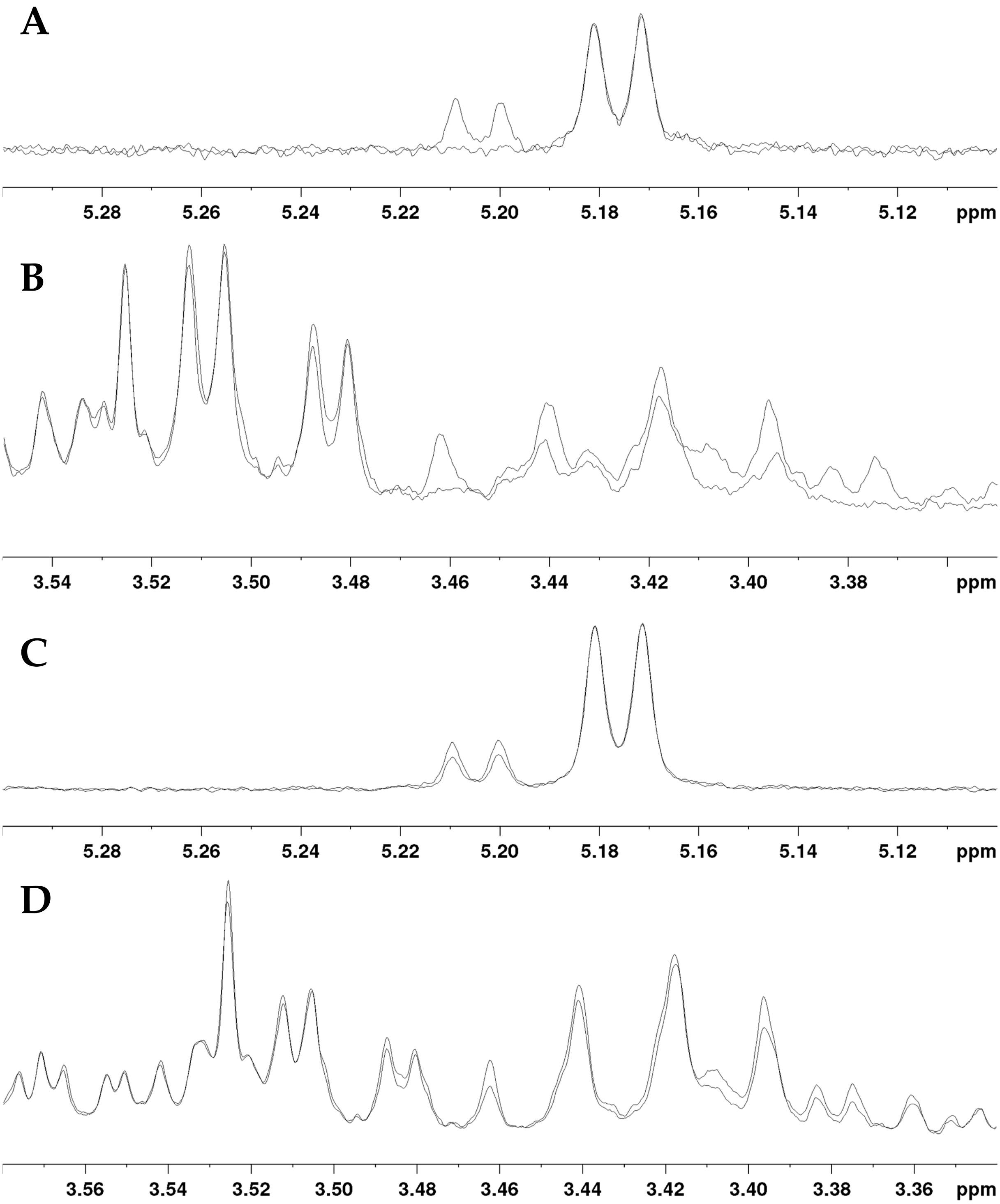

Figure S24: Results of spike-in experiment with 40 µg Glucose at 400 MHz. A: 5.30-5.10 ppm in *T. aestivum*, B: 3.55-3.25 ppm in *T. aestivum*, C: 5.30-5.10 ppm in *T. magnatum*, D: 3.58-3.34 ppm in *T. magnatum*.

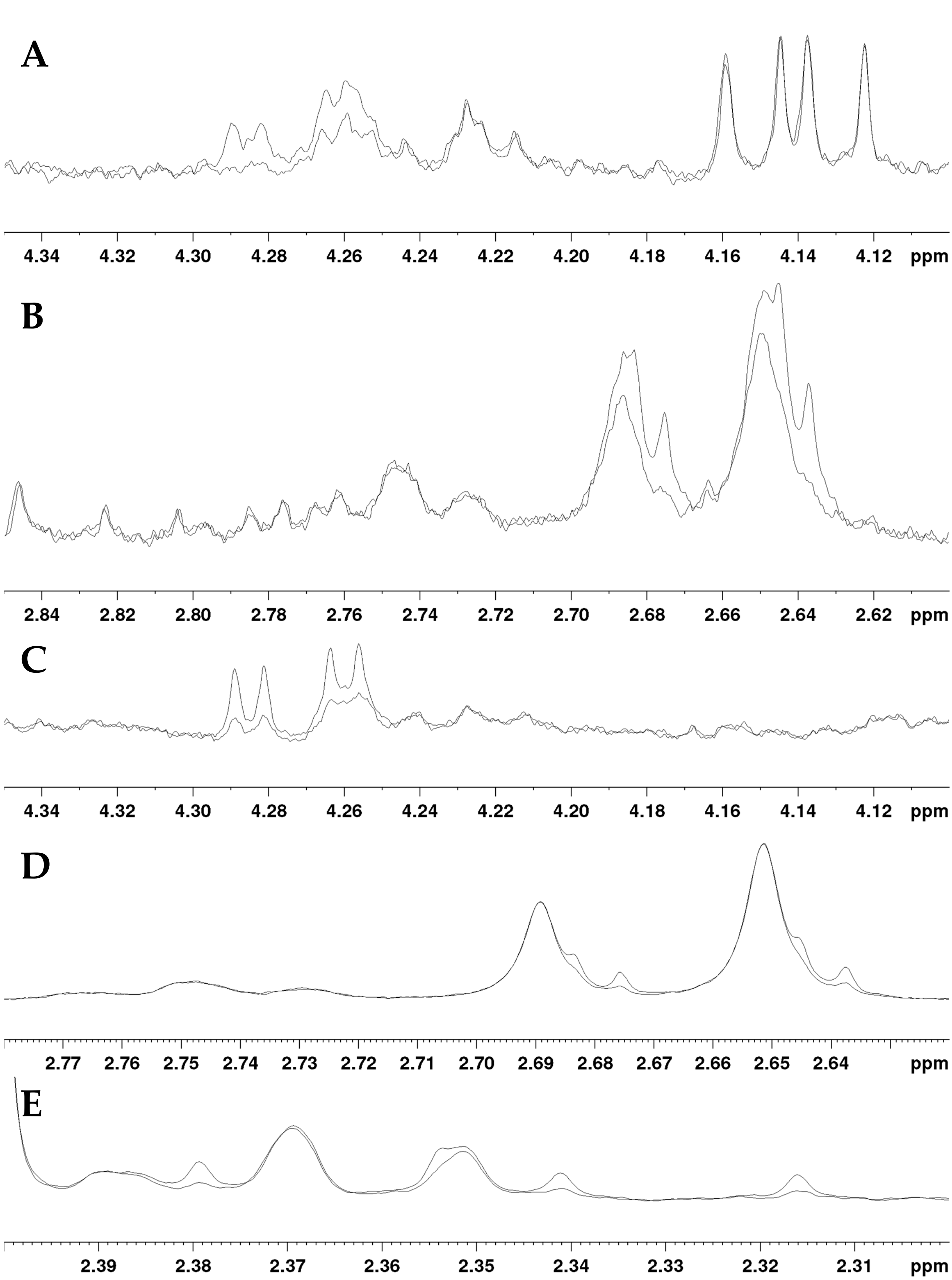

Figure S25: Results of spike-in experiment with 40 µg Malic Acid at 400 MHz. A: 4.35-4.10 ppm in *T. aestivum,* B: 4.35-4.10 ppm in *T. aestivum,* C: 4.35-4.10 ppm in *T. magnatum,* D: 2.78-2.62 ppm in *T. magnatum,* E: 2.40-2.30 ppm in *T. magnatum*.

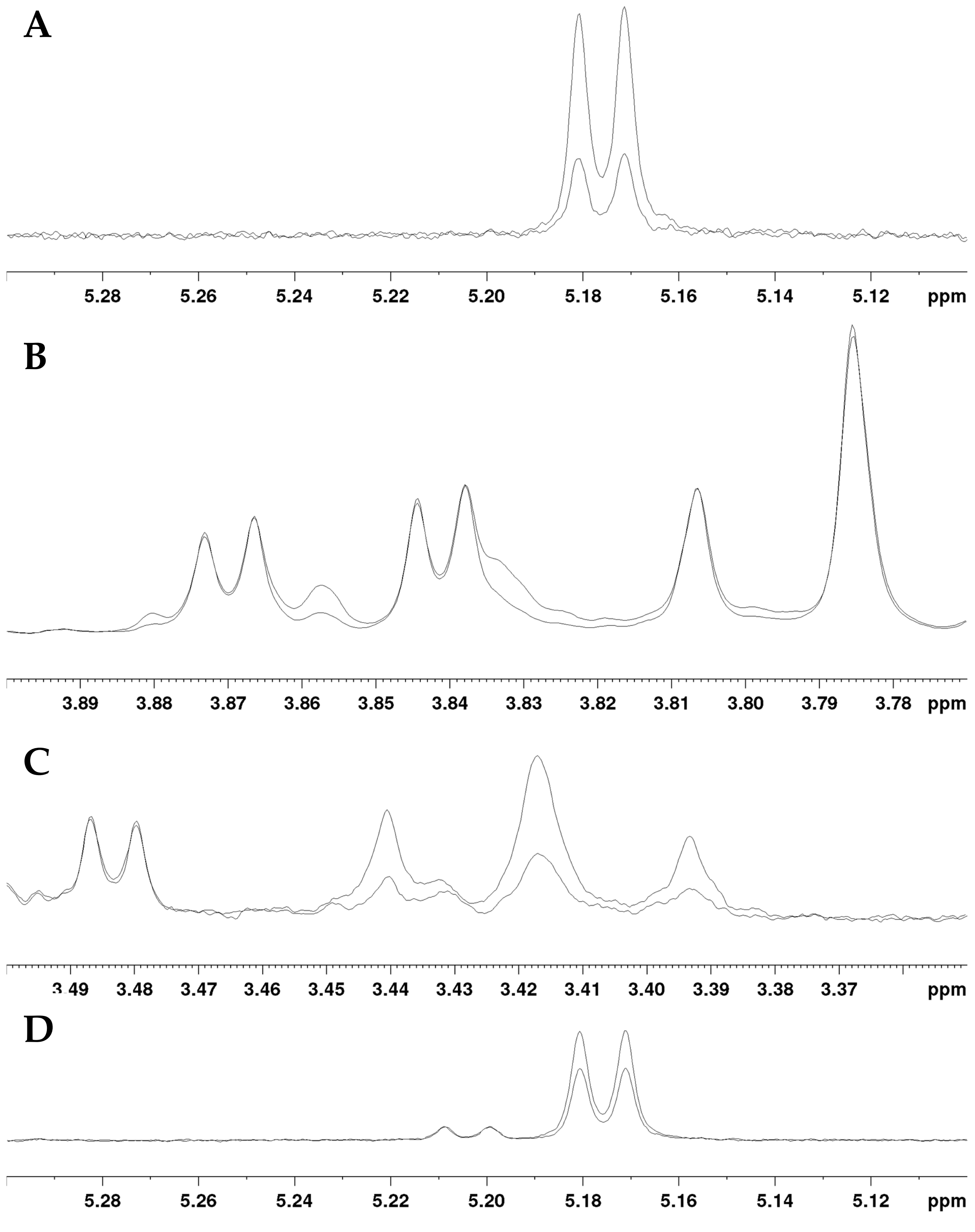
A
5.28 5.26 5.24 5.22 5.20 5.18 5.16 5.14 5.12 ppm
B
3.89 3.88 3.87 3.86 3.85 3.84 3.83 3.82 3.81 3.80 3.79 3.78 ppm
C
3.49 3.48 3.47 3.46 3.45 3.44 3.43 3.42 3.41 3.40 3.39 3.38 3.37 ppm
D
5.28 5.26 5.24 5.22 5.20 5.18 5.16 5.14 5.12 ppm

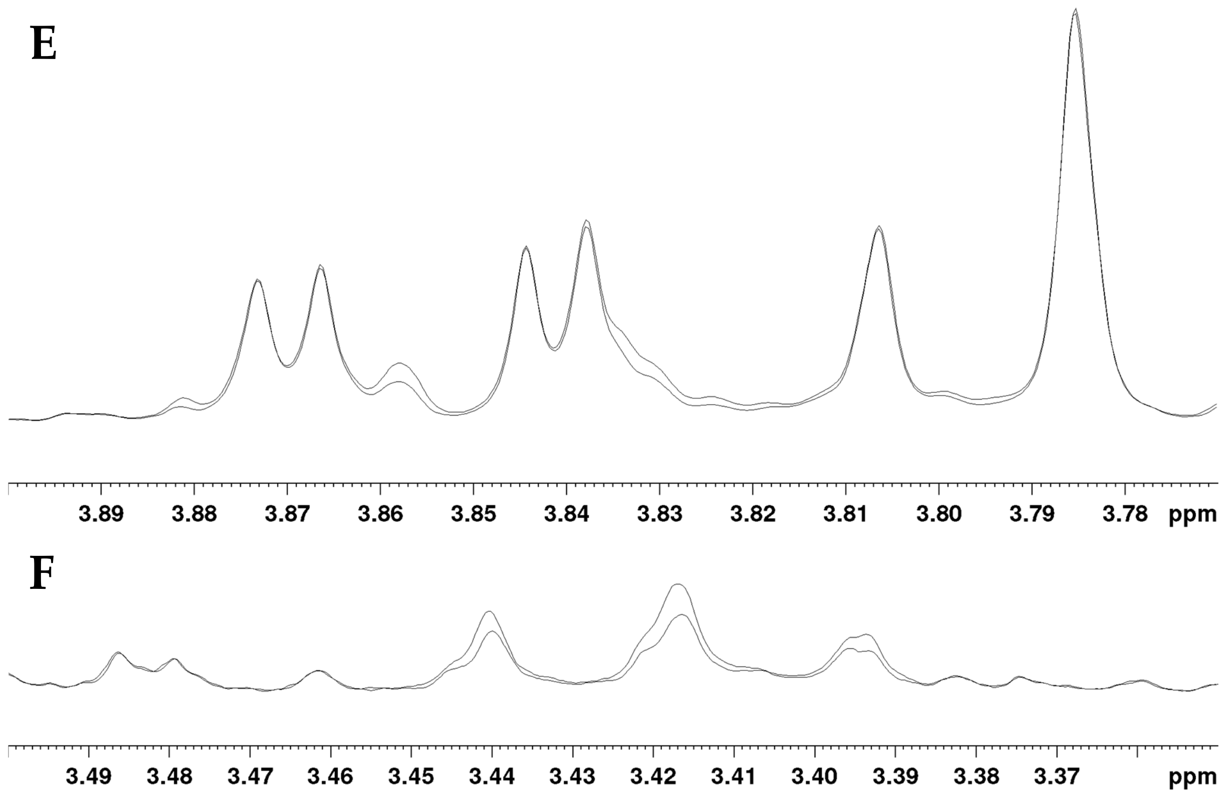

Figure S26: Results of spike-in experiment at 400 MHz with 100 µg Trehalose A: 5.30-5.10 ppm in *T. aestivum*; B: 3.90-3.77 ppm in *T. aestivum*, C: 5.50-3.35 ppm in *T. aestivum*, D: 5.30-5.10 ppm in *T. magnatum*, E: 3.90-3.77 ppm in *T. magnatum*, F: 3.50-3.35 ppm in *T. magnatum*.

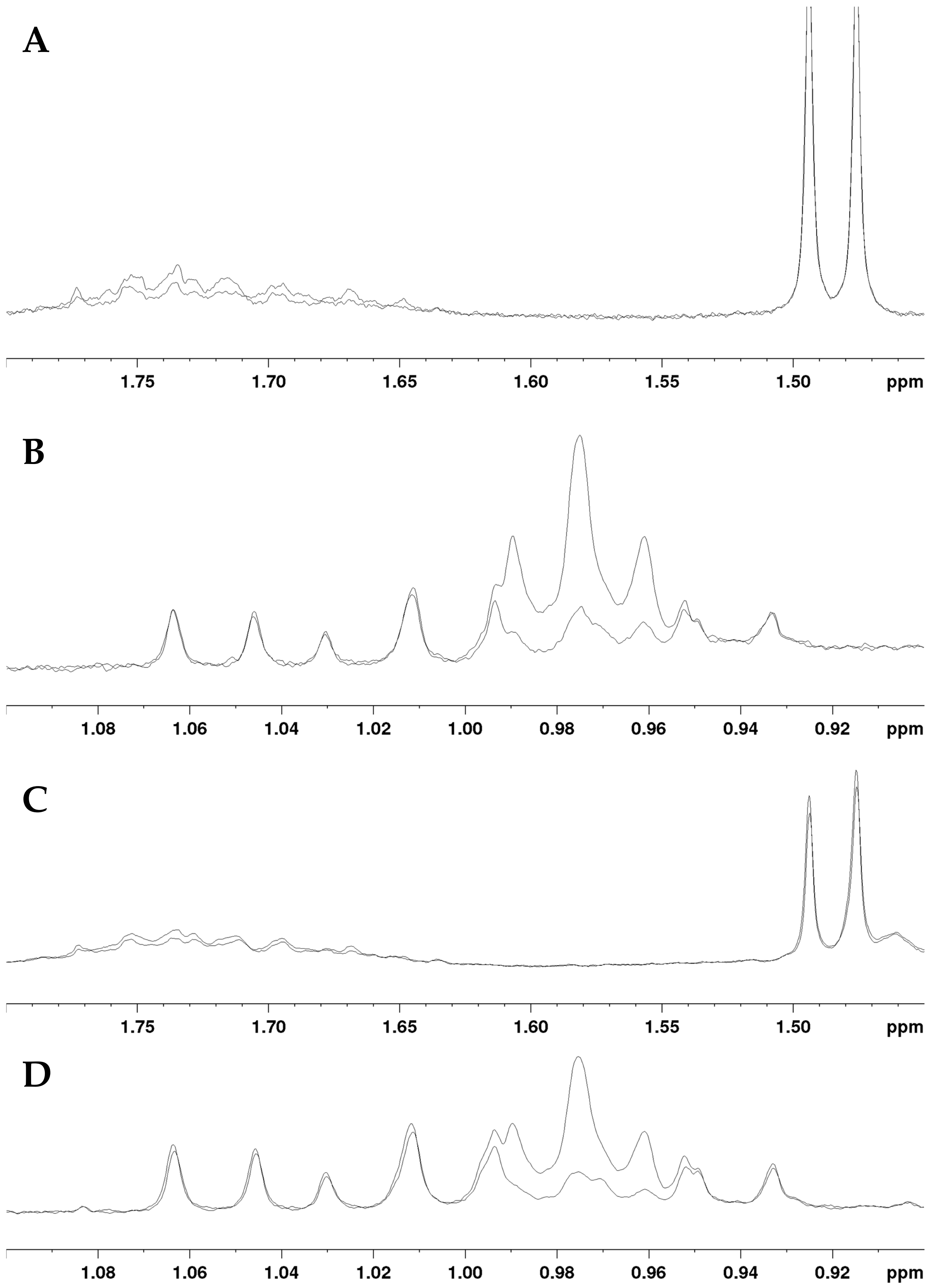

Figure S27: Results of spike-in experiment with 20 µg L-Leucine at 400 MHz. A: 1.80-1.45 ppm in *T. aestivum*, B: 1.09-0.91 ppm in *T. aestivum*, C: 1.80-1.45 ppm in *T. magnatum*, D: 1.09-0.91 ppm in *T. magnatum*.

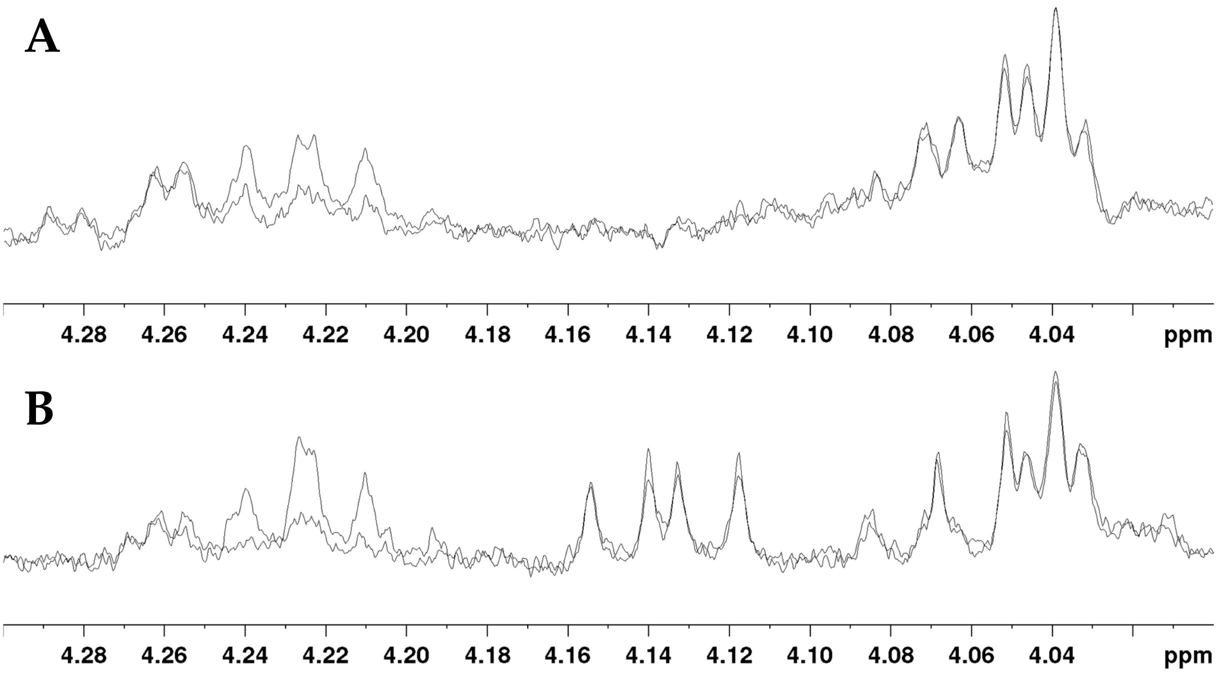

Figure S28: Results of spike-in experiment with 20 µg L-Threonine between 4.30-4.00 ppm at 400 MHz. A: *T. magnatum*, B: *T. aestivum*.

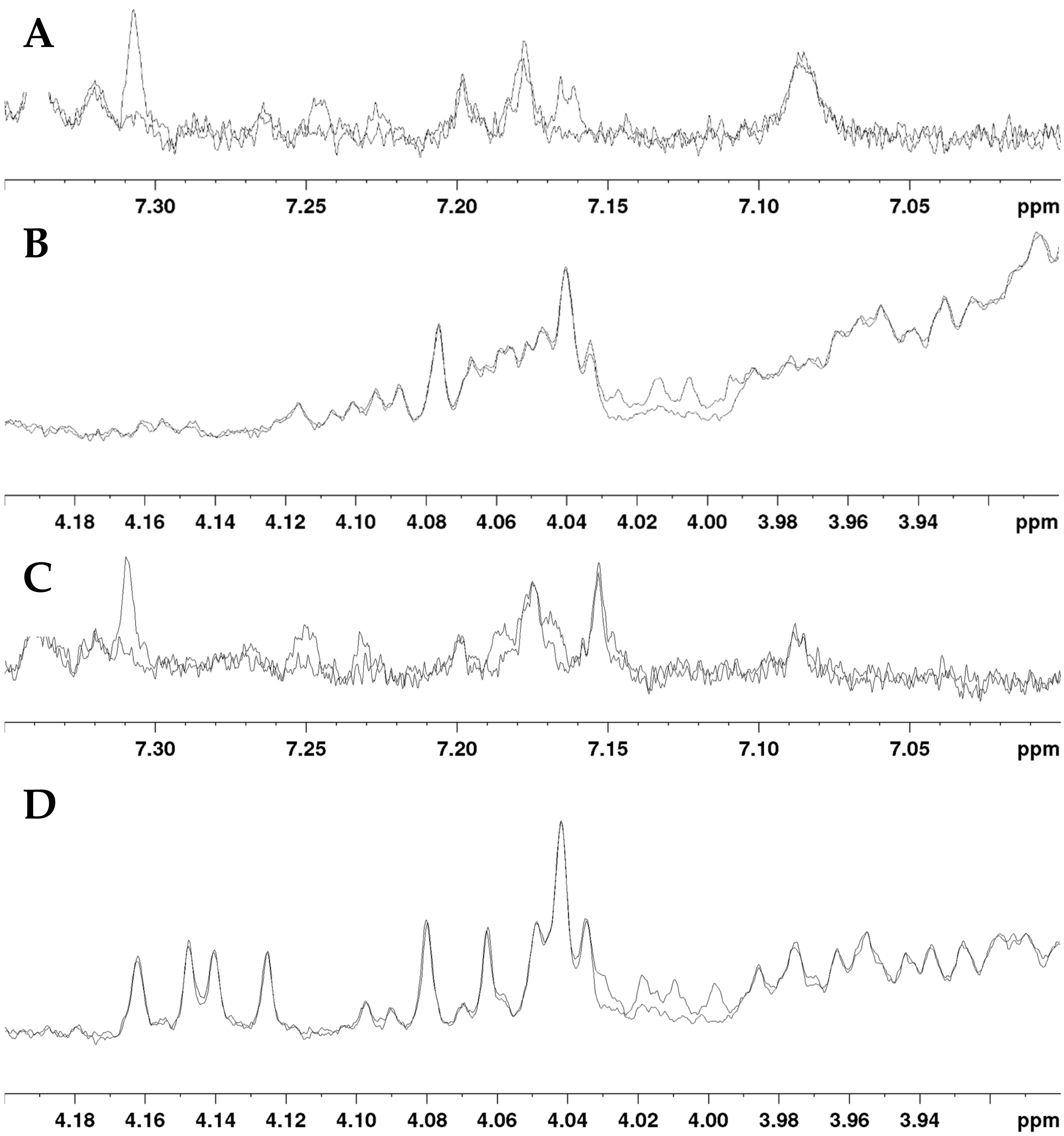

Figure S29: Results of spike-in experiment with L-Tryptophan at 400 MHz. A: 7.35-7.00 ppm with 10 µg in *T. magnatum,* B: 4.20-3.90 with 10 µg in *T. magnatum,* C: 7.35-7.00 ppm with 40 µg in *T. aestivum,* D: 4.20-3.90 ppm with 40 µg in *T. aestivum*.

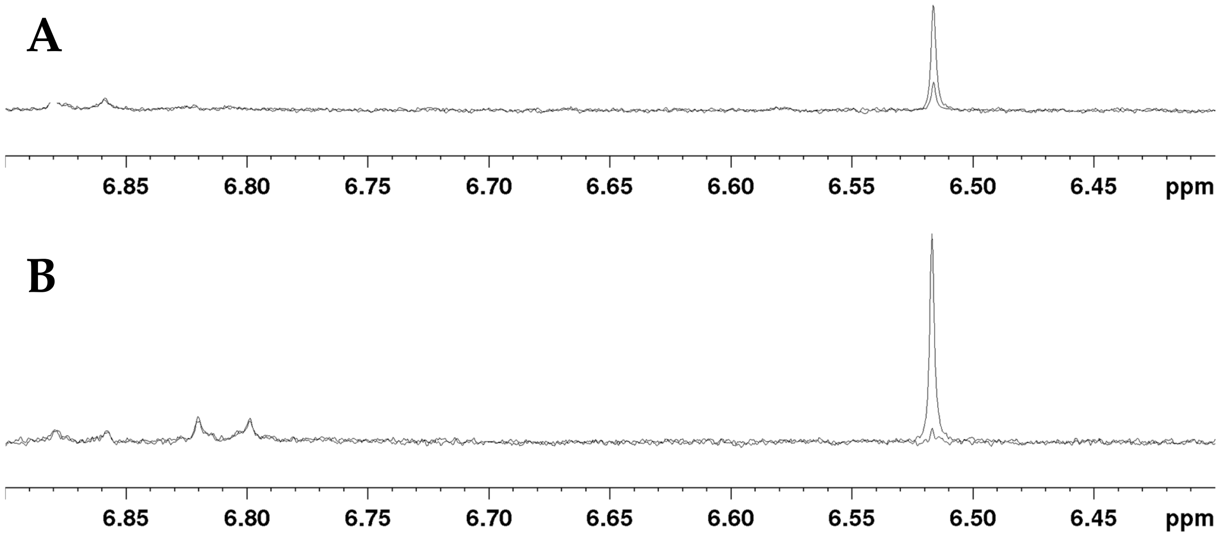

Figure S30: Results of spike-in experiment with Fumaric Acid at 6.90-6.40 ppm at 400 MHz. A: 10 µg in *T. magnatum*, B: 20 µg in *T. aestivum*.

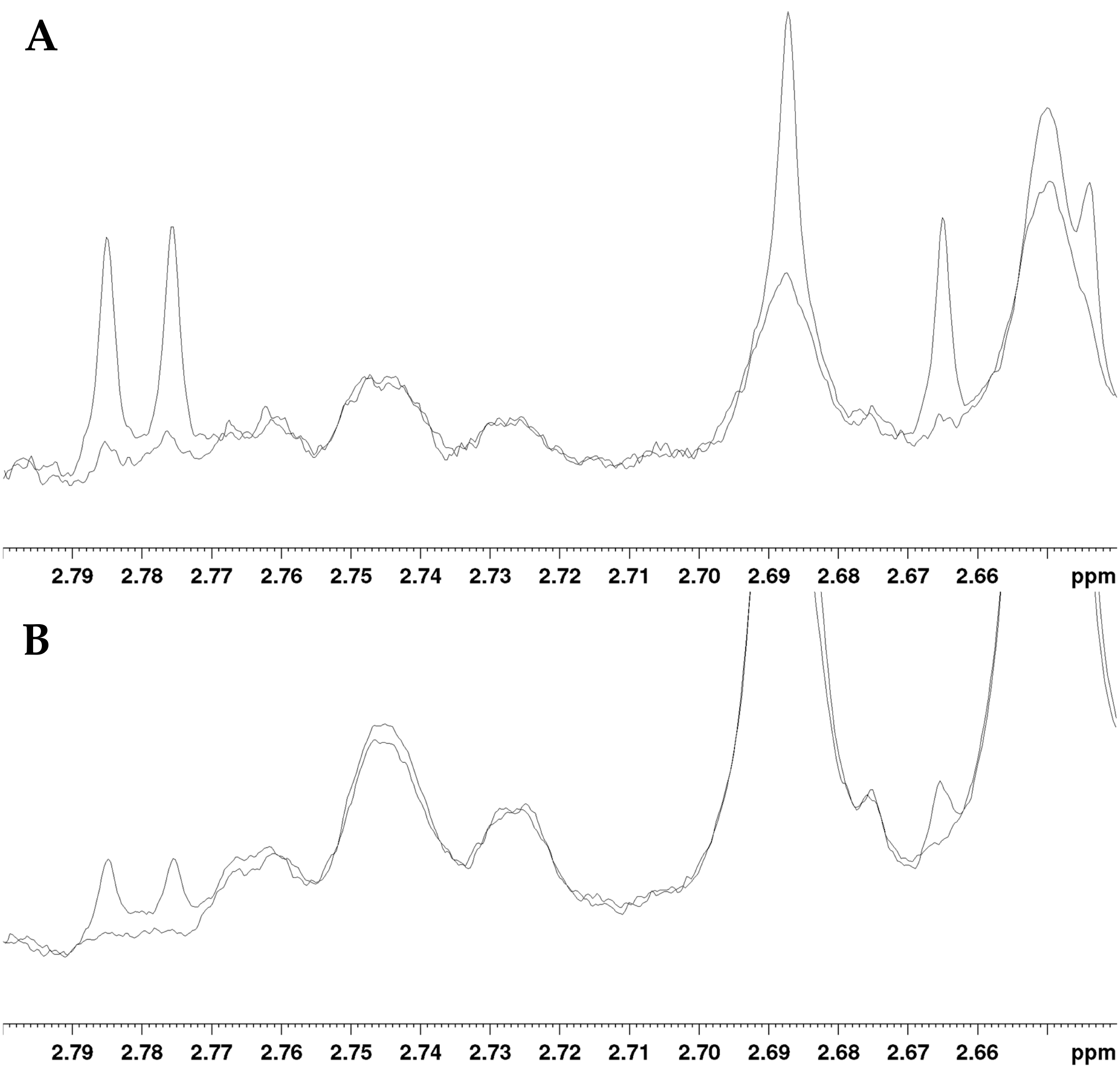

Figure S31: Results of spike-in experiment with L-Aspartic Acid at 2.80-2.62 ppm at 400 MHz. A: 40 µg in *T. aestivum*, B: 20 µg in *T. magnatum* at 400 MHz.

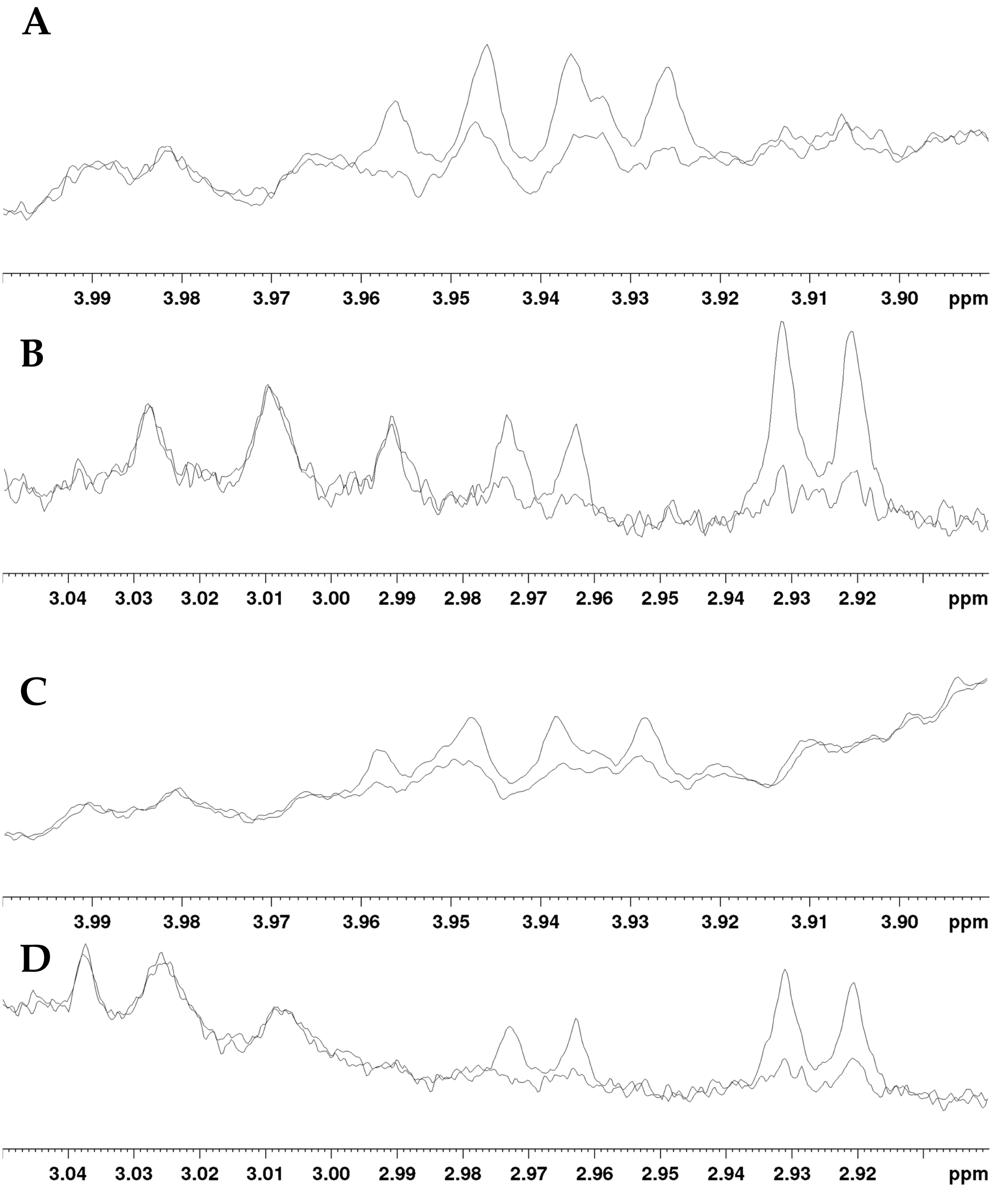

Figure S32: Results of spike-in experiment with 20 µg L-Asparagine at 400 MHz. A: 4.00-3.89 ppm in *T. aestivum,* B: 3.05-2.90 ppm in *T. aestivum,* C: 4.00-3.89 ppm in *T. magnatum,* D: 3.05-2.90 ppm in *T. magnatum.*

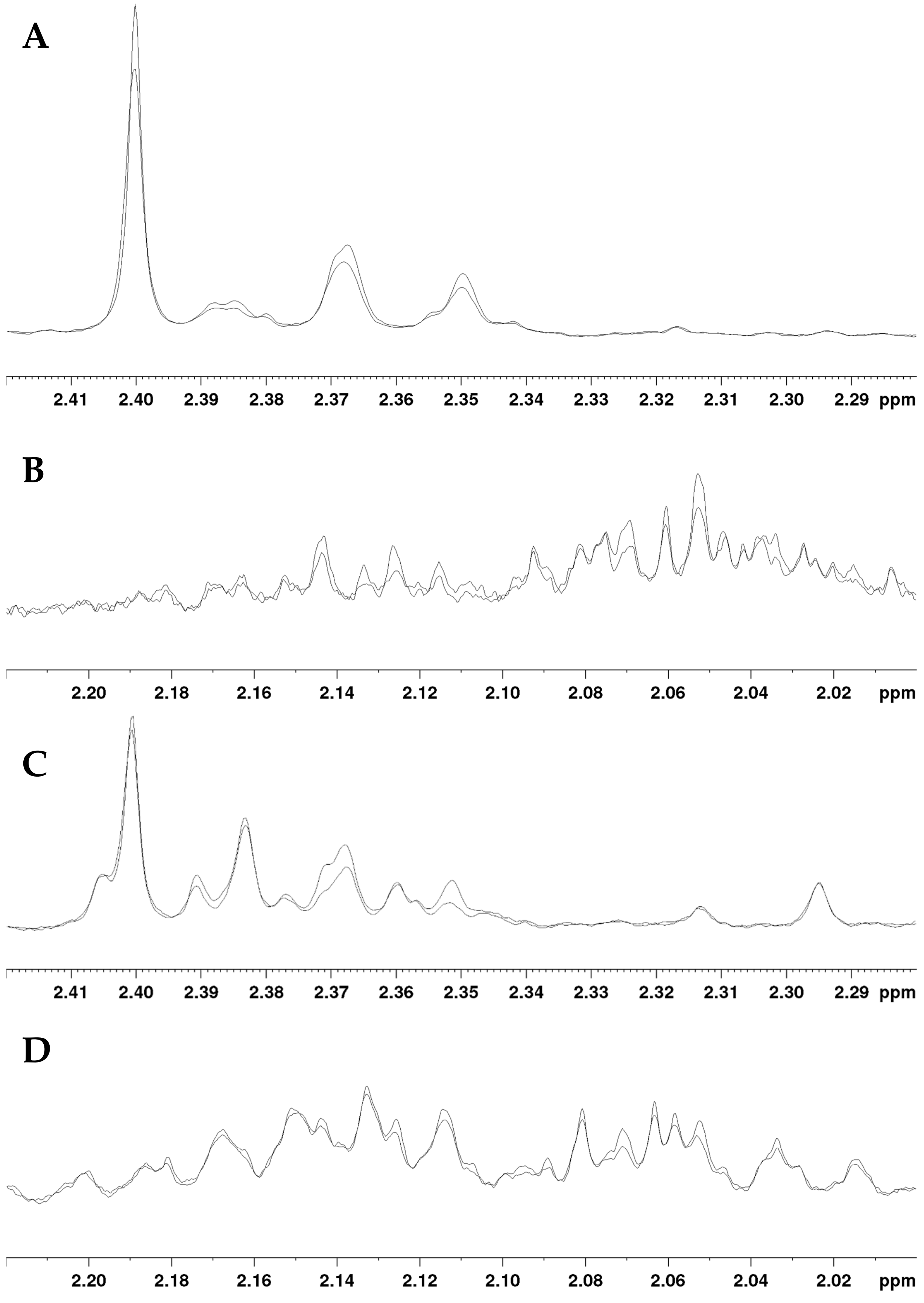

Figure S33: Results of spike-in experiment with 40 µg L-Glutamic Acid at 400 MHz. A: 2.42-2.28 ppm in *T. aestivum,* B: 2.21-2.00 ppm in *T. aestivum,* C: 2.42-2.28 ppm in *T. magnatum*, D: 2.21-2.00 ppm in *T. magnatum*

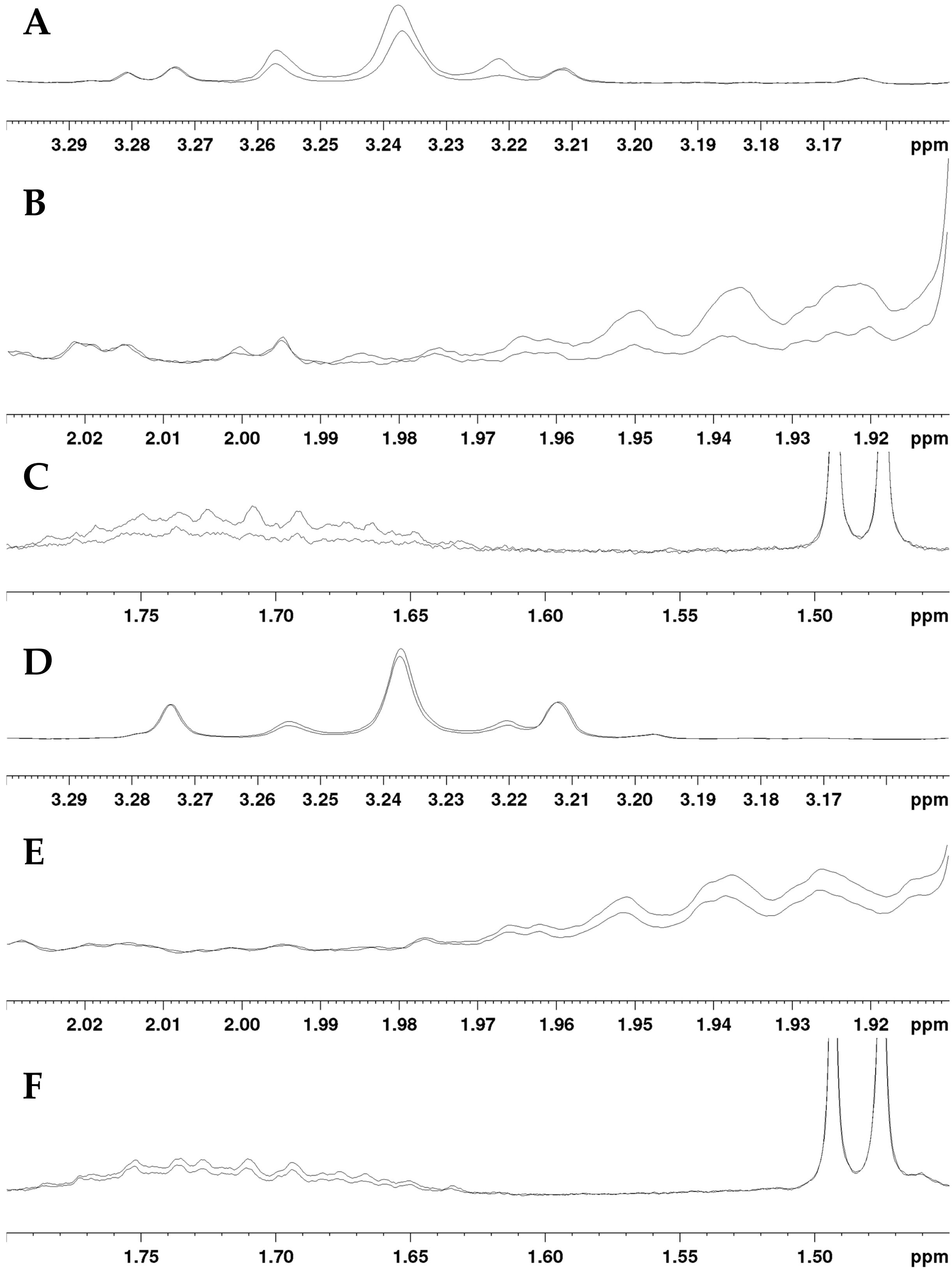

Figure S34: Results of spike-in experiment with 100 µg L-Arginine at 400 MHz. A: 3.30-3.15 ppm in *T. aestivum,* B: 2.03-1.91 ppm in *T. aestivum,* C: 1.80-1.45 ppm in *T. aestivum,* D: 3.30-3.15 ppm in *T. magnatum,* E: 2.03-1.91 ppm in *T. magnatum,* F: 1.80-1.45 ppm in *T. magnatum*.
